# Rapid photo-crosslinking in living cells reveals protein–nucleic acid dynamics on a timescale of minutes

**DOI:** 10.1093/nar/gkag339

**Published:** 2026-04-21

**Authors:** Jakob Trendel, Polina Prokofeva, Zhuo Angel Chen, Lukas Horn, Simon Trendel, Mirea Mema, Marchel Stuiver, Juri Rappsilber, Bernhard Kuster

**Affiliations:** Chair of Proteomics and Bioanalytics, TUM School of Life Sciences, Technical University of Munich (TUM), Freising 85354, Germany; Chair of Proteomics and Bioanalytics, TUM School of Life Sciences, Technical University of Munich (TUM), Freising 85354, Germany; Chair of Bioanalytics, Technical University Berlin, Berlin 10623, Germany; Chair of Proteomics and Bioanalytics, TUM School of Life Sciences, Technical University of Munich (TUM), Freising 85354, Germany; Chair of Proteomics and Bioanalytics, TUM School of Life Sciences, Technical University of Munich (TUM), Freising 85354, Germany; Chair of Bioanalytics, Technical University Berlin, Berlin 10623, Germany; Chair of Bioanalytics, Technical University Berlin, Berlin 10623, Germany; Chair of Bioanalytics, Technical University Berlin, Berlin 10623, Germany; Wellcome Centre for Cell Biology, University of Edinburgh, Edinburgh EH9 3BF, United Kingdom; Chair of Proteomics and Bioanalytics, TUM School of Life Sciences, Technical University of Munich (TUM), Freising 85354, Germany

## Abstract

The activation of chemical reactions in living cells using ultraviolet (UV) light enables the interrogation of biomolecules in their native environment with photoreactive probes or crosslinking reagents. Although numerous photo-crosslinking approaches have been successfully employed, they often suffer from common limitations, including low reaction yields, the need for long exposure times, and irradiation-induced cellular damage from heat, desiccation, or side reactions. We recently showed that 365 nm light-emitting diodes enable rapid, biorthogonal protein–DNA crosslinking in living cells, incurring minimal photodamage. Here, we generalize this approach and demonstrate that high-intensity, longwave UV light reduces the irradiation time for in-cell photo-crosslinking reactions by up to 1000-fold, allowing protein–drug, protein–protein, protein–DNA, and protein–RNA interactions to be fixed within seconds. Benchmarking this rapid photo-activation for the analysis of RNA-interacting proteomes responding to RNA-binding drugs or UV-induced RNA damage, we demonstrate both qualitative and quantitative advantages of controlled, high-intensity UV irradiation, uncovering emergent experimental opportunities that were previously inaccessible to light-activated chemistry in intact cells and tissues.

## Graphical Abstract

**Graphical Abstract:**
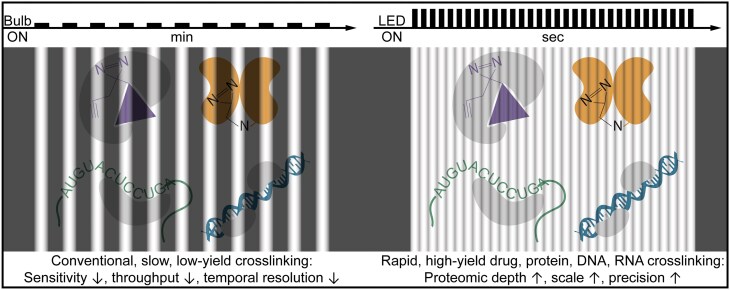

## Introduction

**Figure 1.**
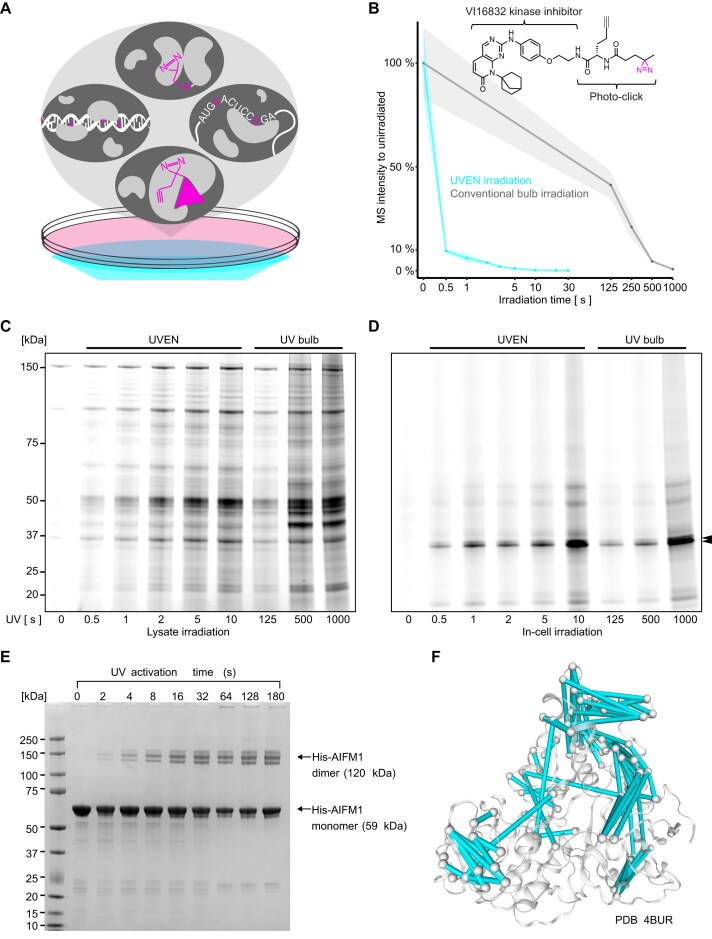
Rapid activation of crosslinking reactions using high-intensity UV irradiation. (**A**) Schematic representation of four photo-crosslinking reactions presented in this study. Photoprobes can be metabolically incorporated into DNA or RNA, or added ectopically in the form of photoreactive drugs or bifunctional protein–protein crosslinkers. Their activation occurs by irradiating culture cells with UV light. (**B**) Line plot showing LC-MS quantification of a kinase inhibitor PAL probe upon irradiation in water. Compared are various irradiation times with a conventional bulb-based device (Vilber Biolink) or the LED-based UVEN. Irradiation occurred at identical distance from the light source (3.5 cm). (**C**) Fluorescence imaging of sodium dodecyl sulfate–polyacrylamide gel electrophoresis (SDS–PAGE) after PAL in native lysates. Compared are various time points of LED irradiation (UVEN) to conventional bulb irradiation at identical distance. (**D**) Same as in panel (B) but for PAL in living cells. Arrows indicate expected molecular weight of CDK4/6 (34/37 kDa). (**E**) SDS–PAGE analysis of sulfo-SDA crosslinked AIFM1 with UV activation for 0–180 s by UVEN. A crosslinked dimeric AIFM1 band appears exclusively upon UV exposure. The yield of the dimeric crosslinked product increased with activation time and plateaued at 32 s. (**F**) Visualization of sulfo-SDA crosslinks obtained after 32 s of UV activation, mapped onto the crystal structure of AIFM1 (PDB ID: 4BUR). A total of 86 crosslinks (87% of 99 displayable crosslinks) with Cα–Cα distances below 30 Å are shown as cyan lines connecting the Cα atoms of crosslinked residues.

The interaction of a biomolecule with a second biomolecule or a drug contains information that can indicate their relationship and function. For example, the interaction of a protein with DNA [1–5], RNA [1, 6–12], or another protein [13, 14] can indicate the biological function of that protein as part of a complex or phase-separated compartment. Similarly, the binding of a kinase or HDAC inhibitor to a specific protein can imply that the molecule inhibits the enzymatic function of this protein. [15, 16] Such an interaction typically occurs through combinations of many weak, non-covalent interactions between the participating molecules. To generate biochemical evidence for this interaction in living cells, photo-crosslinking can be applied (Fig. 1A). To this end, a photoreactive group is introduced into one of the interaction partners, which, upon activation by light, creates an artificial, covalent bond between the two molecules. This photo-crosslinked complex can be purified from non-crosslinked, unspecific interactors using one of the complex partners as enrichment handle. The composition of the complex can subsequently be determined by proteomic, genomic, or transcriptomic analysis. [2, 8–10, 17, 18] Because the linkage between the photo-crosslinked partners is covalent, purification of the complex can be strongly denaturing, leading to very effective elimination of unspecific or indirect interactors. Moreover, the covalent crosslinking site itself can lead to aberrations in the detection of protein or nucleic acids by mass spectrometry or sequencing, respectively, oftentimes pinpointing the exact interaction site in their sequence. [1, 19–22]. Activation of a crosslinking reaction by light is conceptually powerful for cell biology because it is minimally invasive and can be timed. For example, for investigating protein–RNA interactions in culture cells, their transcriptome can be metabolically labeled with the photo-activatable nucleotide 4-thiouridine (4SU). Additional perturbations can be applied to investigate if proteins change their interaction with RNA between conditions and crosslinking is triggered by 1–2 min of irradiation with ultraviolet (UV) bulbs placed directly above the cells. Thus, compared to the most commonly applied form of chemical crosslinking with formaldehyde, which requires 10 min of fixation at room temperature, photo-crosslinking is fast and carried out at 4°C on ice, “freezing” the state of the cell in the moment of crosslinking. Yet, effective protein crosslinking with other photoprobes, such as diazirine-modified small molecules, can require 10–20 min of irradiation or longer [23], and some photoprobes, such as the deoxyribonucleotide 4-thiothymidine, have such low reactivity that conventional bulb-based irradiation systems are often not sufficient. [18]. Here, we show that high-intensity UV irradiation from an LED-based device accelerates commonly used photoreactions in cells by several orders of magnitude, enabling us to perform protein–drug, protein–protein, protein–DNA, and protein–RNA crosslinking within seconds while maintaining cells in their original medium. Under physiological culture conditions, we further demonstrate quantitative and qualitative advantages of high-intensity photoactivation and apply rapid protein–RNA crosslinking to profile RNA-interacting proteomes in breast cancer cells exposed to a panel of RNA-binding drugs. Finally, we exploit the fast RNA crosslinking kinetics to induce targeted RNA damage and quantify the resulting changes in cytosolic protein–RNA interactions, revealing very early engagement of the ribosome rescue machinery and activation of the ribotoxic stress response.

## Materials and methods

### Cell culture

MCF7 (human female breast adenocarcinoma) and U2OS (human female bone sarcoma) were obtained from ATCC. U251 (human male glioblastoma) and HT-29 (human female colon carcinoma) were obtained as part of the NCI60 panel. Cells were maintained in Dulbecco’s modified Eagle’s medium (DMEM) supplemented with 10% dialyzed FBS and Pen–Strep (100 U/ml penicillin, 100 µg/ml streptomycin) at 37°C, 5% CO_2_. For the comparative analysis of DNA interactomes, 2 million cells were seeded and expanded in the presence of 100 µM 4-thiothymidine (4ST) over four days. For the comparative analysis of RNA interactomes, 2 million cells were expanded for three days and 100 µM 4SU added for 24 h before UV crosslinking.

### Construction of the UVEN irradiation device

Housings of various prototypes were built from milled wood and 3D-printed plastic elements, which served as scaffold for the assembly of commercially available aluminum and electrical parts. High-intensity UV-LEDs (16 Luminous SBM-120-UV-F34-H365-22) were soldered on a custom copper-based PCB and mounted onto an aluminum heat sink. A publication on the construction of the device is currently in preparation. UVEN is presented as an open-science project, for which we provide detailed bills of materials, computer-aided designs, and construction manuals as well as open-source firmware at www.uven.org.

### Operation of the UVEN irradiation device for UV crosslinking

For UV activation of photo-crosslinking reactions in cells cultured on 15 cm diameter culture dishes, the UVEN irradiation device was operated at 3.5 A per LED, resulting in 2000 ± 300 mW/cm^2^ intensity across the entire area of the culture dish. For protein–DNA crosslinking, 30 s of UVEN irradiation were applied, where cells were washed with 50 ml ice-cold PBS and subsequently irradiated in another 50 ml PBS. For all other experiments with irradiation times up to 5 s, cells were placed from the incubator immediately into the irradiation device, remaining in their original medium. For the analysis of the cytosolic RNA-interacting proteome after RNA photodamage, 4SU-labeled MCF7 cells were irradiated for 1 s in their original medium, followed by another 5 s pulse immediately after or 120 s later, while cells remained in the UVEN device.

### In-cell photo-affinity labeling and click chemistry

Palbociclib photo-affinity labeling (PAL) probe was synthesized in a single-pot reaction without further purification using an NHS-activated minimalist photo-click compound (CAS 2012552-32-6, Enamine, EN300-28319299). To this end, 10 µl of 100 mM palbociclib in water (CAS 571190-30-2, MedchemExpress, HY-50767) was mixed with 11 µl of 100 mM photo-click freshly prepared in water, 1 µl of triethylamine was added, and the mixture was incubated at 20°C and 800 rpm shaking for 20 h. Completion of the reaction was analyzed by LC-MS, typically confirming >99% conversion. Palbociclib-PAL probe was added at 1 µM concentration from a 1 mM stock in DMSO to the media of 70%–80% confluent HeLa cells growing on a 10 cm culture dish and normal cell culture continued for 1 h. Cells were transferred into the UVEN device and immediately irradiated for 0.5–10 s in their original media. For bulb irradiation at a comparable distance, the device was inverted, and culture dishes were propped up on both sides with dedicated placeholders at a distance of 3.5 cm from the UV bulbs, allowing irradiation for 125–10 000 s while the cells remained in their original media. In both cases, cells were washed twice with 10 ml ice-cold PBS, which was removed to completion. Cells were scraped into 500 µl ice-cold, denaturing lysis buffer (25 mM HEPES pH = 7.5, 150 mM NaCl, 2 mM MgCl_2_, 0.1% NP40, 8 M urea) and sonicated three times for 10 s with a Sonopuls ultrasonic homogenizer mini20 (BANDELIN) at 50% energy on ice with thorough vortexing and spinning down in between. Lysates were centrifuged at 4°C with 15 000 × *g* for 15 min and supernatants transferred to fresh tubes. The protein content was determined by BCA assay and protein concentrations adjusted to 1 mg/ml with denaturing lysis buffer. Fluorescent dye Cy5.5-azide (Jena Bioscience) was clicked to 50 µg protein lysate by addition of 2 µM Cy5.5-azide, 5 mM CuSO4, 5 mM THPTA, 10 mM sodium ascorbate, and 10 mM aminoguanidine for 2 h at 20°C and 800 rpm shaking. To remove excess salts for gel analysis samples were acetone precipitated and pellets resolubilized in sodium dodecyl sulfate–polyacrylamide gel electrophoresis (SDS–PAGE) running buffer. Native lysates were produced from untreated HeLa cells washed with ice-cold PBS, which was removed to completion. Cells were scraped into 200 µl ice-cold, native lysis buffer (25 mM HEPES pH = 7.5, 150 mM NaCl, 2 mM MgCl_2_, 0.1% NP40), sonicated, centrifuged, and adjusted to 10 mg/ml with native lysis buffer as described earlier. Pre-clicked palbociclib-PAL-Cy5.5 conjugate was added to a final concentration of 1 µM into 25 µg of HeLa native lysate in 10 µl reaction volume and incubated at 20°C for 30 min with 300 rpm shaking. Tubes were irradiated with UV at a distance of 3.5 cm from the light source (identical distance to LED or bulb). For SDS–PAGE analysis approx. Ten micrograms of HeLa protein was resolved on a Bis-Tris 4%–12% gradient gel (Invitrogen) and imaged on an Odyssey IR scanner (LI-COR).

### *In vitro* crosslinking of AIFM1

The gene encoding apoptosis-inducing factor, mitochondrial (AIFM1), with an N-terminal 6× His tag was expressed in *Escherichia coli* strain BL21 [24]. Cells were harvested and resuspended in buffer containing 50 mM Tris–HCl (pH 7.8), 500 mM NaCl, 4 mM MgCl₂, 0.5 mM tris(2-carboxyethyl)phosphine (TCEP), 30 mM imidazole, and protease inhibitors (Roche). Cells were lysed by sonication on ice. Debris was removed by centrifugation, and the protein was purified by Ni-NTA affinity chromatography. For further purification, size-exclusion chromatography was performed using a HiLoad 16/600 Superdex 200 pg column (GE Healthcare) equilibrated with 10 mM Tris–HCl (pH 7.8), 150 mM NaCl, and 0.5 mM TCEP. The eluted protein was concentrated to 52 mg/ml, then rebuffered into crosslinking buffer (10 mM HEPES-NaOH, pH 7.8, 150 mM NaCl, 4 mM MgCl₂, 0.5 mM TCEP) and diluted to 1 mg/ml. To induce dimerization, the sample was incubated on ice with 1 mM NADH for 1 h. The crosslinking reaction was performed similarly to previous reports [25]. Sulfo-SDA was freshly prepared in crosslinking buffer at 2 mM. Nine separate 23 µg aliquots of AIFM1 (1 mg/ml) were incubated on ice with 0.5 mM sulfo-SDA for 1 h. To quench the NHS-ester reaction, 50 mM Tris–HCl was added and incubated for 15 min. Each 30 µl sample was pipetted into the center of a well in a Greiner Bio-One polystyrene 24-well cell culture plate and irradiated with UV light using the UVEN device at maximum intensity for 0, 2, 4, 8, 16, 32, 64, 128, and 180 s. The crosslinked protein samples were then separated by NuPAGE™ 4%–12% Bis-Tris gel electrophoresis using MOPS buffer at 180 V. Bands corresponding to monomeric and dimeric AIFM1 (from the 32-s activation sample) were excised and digested in-gel with trypsin, as previously described [26]. The resulting peptides were desalted using C18 StageTips [27] prior to LC-MS/MS analysis.

### In-cell crosslinking with L-photo-leucine

The stable HEK293 expressing Rpn11-HTBH cell line (ABM) that incorporates L-photo-leucine [28] was grown in 145 mm diameter dishes (Greiner) in DMEM (high glucose, 10% FBS). Cells growing with normal L-leucine instead of L-photo-leucine were used as a control. For harvesting, cells were washed twice with warm PBS and 10 ml ice-cold PBS was added to the plates. L-photo-leucine activation occurred with UVEN by irradiating twice at maximum intensity for 10 s, with a 10-s pause in between. Cells were scraped, centrifuged at 200 × *g* for 5 min, and boiled in Laemmli buffer. Samples were run on 4%–20% Mini-PROTEAN TGX Precast protein gels (Bio-Rad) and blotted on PVDF membranes (Bio-Rad). After blocking the blot in 5% BSA in TBST, incubating for 30 min with streptavidin-HRP (1:20000, Thermo Fisher), and washing six times with TBST, the biotinylated signals were detected using SuperSignal West Pico PLUS Chemiluminescent Substrate (Thermo Fisher) using a Bio-Rad ChemiDoc XRS+ system.

### Extraction of protein-crosslinked DNA (XDNAX)

The extraction was performed similar to that described recently with slight variation [29]. In brief, cell pellets from one confluent 10 cm diameter culture dish were lysed in 1 ml TRIZOL (T9424, Sigma–Aldrich) by pipetting until completely homogenous, combined with 200 µl chloroform, and centrifuged at 12 000 × *g* for 10 min at 4°C. The aqueous phase was discarded and the interphase transferred to a fresh tube, and TRIZOL lysis repeated a second time. The aqueous phase was discarded and the interphase transferred to a fresh tube to be disintegrated in 1 ml recovery buffer [Tris–Cl (pH 7.5, 50 mM), ethylenediaminetetraacetic acid (EDTA) 1 mM, SDS 1%] until completely dissolved. The DNA was isopropanol precipitated and rehydrated in 900 µl of water with 0.1% SDS. RNA was digested with 0.5 µg RNase A and T1 for 60 min at 37°C, 700 rpm shaking. DNA was fragmented by Covaris sonication in 120 µl portions using the parameters 50 cycles, scan speed: 1.0, PIP: 300, CPB: 50, AIP: 75, dithering: Y = 1, speed = 10. SDS concentration was adjusted to 2%, and the sample was incubated at 95°C for 5 min with 700 rpm shaking. The sample volume was doubled by addition of 5 M guanidinium thiocyanate (GuTC) and again incubated at 95°C for 5 min with 700 rpm shaking. The sample was applied to a silica spin column (Zymo-Spin III CG Column) and washed twice with 800 μl wash solution (GuTC 2.5 M, ethanol 50 %), and three times with 800 μl ethanol 70 %, each time centrifuging 2 minutes with 10000 g. Crosslinked protein–DNA complexes were eluted from the column overnight with 300 µl nuclease elution mix [NEB nuclease P1 (NEB) buffer 1×, MgCl_2_ 5 mM, 0.5 µl NEB nuclease P1, 0.5 µg benzonase (Santa Cruz)] and again eluted with 300 µl elution buffer (Tris–Cl 50 mM, SDS 2%). For protein cleanup, 10 µl of SP3 beads (GE44152105050250, Sigma–Aldrich, original slurry) were added and vortexed before addition of 1 ml of 100% ethanol, mixing, and protein aggregation for 15 min. The beads were washed four times with 2 ml of 70% ethanol and subsequently spun down to remove all residual ethanol. Protein was digested off the beads overnight in 200 µl trypsin digestion buffer (EPPS 50 mM pH = 8, 5 mM DTT, 0.5 µg trypsin per sample) at 37°C, 700 rpm shaking. Cysteines were alkylated by addition of 6 µl chloroacetamide 550 mM while the incubation was continued for 60 min. The beads were collected on a magnet for 5 min and the supernatant transferred to a fresh vial. Peptides were cleaned up by SCX StageTip followed by C18 StageTip and fractionation into six high-pH fractionations using 250 mM ammonium formate buffer (pH = 10) containing 0, 5, 10, 15, 20, and 50% acetonitrile. Fractions with 5% and 50%, as well as 0% and 20% acetonitrile, were combined, and samples were analyzed in four final fractions.

### Extraction of protein-crosslinked RNA (XRNAX)

An improved variation of the previously reported purification was applied [9], which exclusively quantifies on protein level and refrains entirely from trypsin predigestion. After photocrosslinking, MCF7 cell pellets from one confluent 15 cm diameter culture dish were lysed in 1 ml TRIZOL by pipetting until completely homogenous, combined with 200 µl chloroform, and centrifuged at 12 000 × *g* for 10 min at 4°C. The aqueous phase was discarded and the interphase transferred to a fresh tube to be disintegrated in 900 µl recovery buffer (50 mM Tris–Cl pH = 7.5; 1 mM EDTA, 1% SDS) until completely dissolved. Samples were combined with 100 µl NaCl 5 M and 1 ml isopropanol, mixed, and incubated at −20°C for 30 min. The precipitated sample was spun for 15 min at 17 000 × *g* at −11°C. The resulting pellet was washed with 2 ml of 100% EtOH and ethanol residues removed to completion. The pellet was then detached from the wall and rehydrated in 430 µl ultrapure water at 4°C for 15 min on a rotating wheel. The hydrated cloud was further dissolved by pipetting before addition of 50 µl DNase I buffer 10× (NEB), 1 µl RNasin, and 20 µl DNase (NEB). DNA was digested for 30 min at 37°C, 700 rpm shaking, remaining clumps dissolved by pipetting, and the digestion resumed for another 15 min. Samples were combined with 1.5 ml Qiagen RNeasy lysis buffer (Qiagen) and heated to 80°C for 10 min at 500 rpm shaking. After they reached room temperature, samples were centrifuged at 12 000 × *g* for 5 min, and 1900 µl transferred to a fresh tube leaving precipitates behind. They were combined with 1 ml of 100% ethanol, mixed, and applied to an RNeasy Midi spin column at 3000 × *g* for 2 min at room temperature. Columns were washed twice with 4 ml buffer RW and three times with buffer RPE. Protein-crosslinked RNA was then eluted twice with 300 µl ultrapure water. RNA was digested overnight at 37°C, 700 rpm shaking, after addition of 30 µl Tris–Cl 1 M along with 0.5 µg of the RNases A (Sigma–Aldrich), T1 (Sigma–Aldrich), benzonase (Santa Cruz), and 5 µl of MgCl_2_ 1 M. For SP3 cleanup of proteins, 100 µl of 20% SDS was added, and samples were denatured at 90°C for 5 min. After reaching room temperature, 10 µl of SP3 beads were added (GE44152105050250, Sigma–Aldrich, original slurry) along with 1 ml of 100% ethanol, and protein aggregation was allowed to occur for 15 min. Beads were collected on a magnet, washed four times with 2 ml of 70% ethanol, and all residual ethanol was removed. Proteins were digested off the beads in 200 µl trypsin digestion buffer (EPPS 50 mM, 5 mM DTT, 0.5 µg trypsin) overnight at 37°C with 700 rpm shaking. For cysteine alkylation, 6 µl of CAA 550 mM were added, and incubation continued for 60 min. Beads were collected on a magnet and 200 µl of the digested peptides mixed with 10 µl of 10% formic acid in a fresh tube. Samples were centrifuged at 20 000 × *g* for 5 min at room temperature, and 200 µl of the acidified peptides transferred to a fresh tube. Peptides were cleaned up by SCX StageTip (225166889-U, Sigma–Aldrich), dried down, and desalted by C18 StageTip (66883-U, Sigma–Aldrich). For the benchmarking of this protocol, we selected a set of RNA-binding compounds, including translation inhibitors (cycloheximide [CHX], harringtonine [HAR], puromycin [PUR]), a series of tetracyclines (tetracycline [TET], doxycycline [DOX], minocycline [MIN], neocycline [NEO]), and splicing/translation modulators (ataluren [ATA], risdiplam [RIS], branaplam [BRA], isoginkgetin [ISO]). All compounds were purchased from Hycultec, except cycloheximide from Sigma–Aldrich. Cells were photolabeled by addition of 100 µM 4SU to the medium for 24 h. All drugs were added to a final concentration of 10 µM from a 1:1000 stock in DMSO. After 5 min of incubation at normal culture conditions, cell culture dishes with media were transferred to the UVEN device, irradiated for 5 s, and cells were immediately harvested on ice.

### Extraction of cytosolic protein-crosslinked RNA (cytosolic XRNAX)

After photocrosslinking, MCF7 cells from one confluent 10 cm diameter culture dish were lysed in 1 ml ice-cold hypotonic nuclear extraction buffer (50 mM Tris–Cl, pH = 7.5; 0.1% NP40; 60 mM KCl; 1.5 mM MgCl_2_) by pipetting 10 times with a 1 ml tip, followed by 10 min of incubation on ice and another intermittent 10 times pipetting. Nuclei were pelleted by centrifugation with 2000 × *g* at 4°C for 10 min, and subsequently 900 µl of the supernatant containing the cytosol was transferred to a fresh 2 ml tube. Samples were combined with 100 µl NaCl 5 M and 1 ml isopropanol, mixed, and incubated at −20°C for 30 min. The precipitated samples were spun for 15 min at 17 000 × *g* at −11°C, and the resulting pellet was washed twice with 1 ml of 70% EtOH. Residual EtOH was removed and the pellet dissolved in 600 µl RNA Lysis Buffer (RNeasy Mini Kit) and heated to 60°C for 10 min. Samples were allowed to reach room temperature before addition of 600 µl of 70% EtOH and mixing by inversion. Samples were applied to the spin columns and washed once with buffer RW1 and twice with buffer RPE. Cytosolic protein-crosslinked RNA was eluted twice with 100 µl ultrapure water. For RNA digestion, 20 µl Nuclease P1 buffer (NEB), 1 µl Nuclease P1 (NEB), as well as 1 µl of 1 M MgCl_2_, and 0.5 µg benzonase (Santa Cruz) were added, and samples were incubated overnight at 37°C with 700 rpm shaking. Subsequently, 400 µl SDS buffer was added (50 mM Tris–Cl, 2% SDS), and samples were denatured at 90°C for 5 min. SP3 cleanup, trypsin digestion, and SCX StageTip occurred as described earlier for XRNAX samples. SCX-cleaned-up peptides were quantified by Nanodrop UV spectroscopy and 0.25 µg loaded on EvoTips (Evosep) before analysis by LC-MS.

### LC-MS/MS analysis of proteomic samples

For protein–DNA and protein–RNA crosslinking samples, analysis via data-dependent acquisition (DDA) occurred on an Orbitrap Eclipse Tribrid mass spectrometer (Thermo Scientific), connected to a Dionex UltiMate 3000 RSLCnano system (Thermo Scientific). Samples were injected onto a trap column (75 µm × 2 cm) packed with 5 µm C18 resin (Dr Maisch Reprosil PUR AQ) in solvent C (formic acid 0.1%). Peptides were washed with solvent C at 5 µl/min for 10 min and subsequently transferred onto an analytical column (75 µm × 48 cm, heated to 55°C) packed with 3 µm C18 resin (Dr Maisch Reprosil PUR AQ) at a flow rate of 300 nl/min using a gradient of solvent from 4% B (formic acid 0.1% in acetonitrile, DMSO 5%) followed by a linear increase to 32% B in A (formic acid 0.1% in ultrapure water, DMSO 5%). Nanosource voltage was 2000 V, and ion transfer tube temperature was 275°C. Detection occurred with DDA using an OT–OT method and a cycle time of 2 s. MS1 resolution was 60 000, scan range 360–1300 m/z, RF lens 40%, AGC target 100%, and maximum injection time 50 ms. MS2 isolation occurred with a quadrupole window of 1.2 m/z and fragmentation with 30% HCD energy. MS2 resolution was 30 000, first mass 100 m/z, AGC 200%, and maximum injection time 54 ms. For protein–DNA (XDNAX) and protein–RNA (XRNAX) crosslinking, analysis via data-independent acquisition (DIA) occurred on a timsTOF HT mass spectrometer (Bruker), connected via a CaptiveSpray source (Bruker) to a Vanquish Neo UHPLC System (Thermo Scientific). Samples were loaded onto an analytical Aurora Ultimate CSI column (75 µm × 25 cm) packed with 1.7 µm C18 resin (IonOpticks, #AUR3-25075C18-CSI) under pressure control at a maximum of 1000 bar. Peptide separation was performed at a flow rate of 250 nl/min using a gradient of solvent from 9% B (formic acid 0.1% in acetonitrile) followed by a linear increase to 28% B within 19.8 min, followed by a linear increase to 37% B within 4.5 min in A (formic acid 0.1% in ultrapure water). An end plate offset of 500 V, capillary voltage of 4500 V, and a dry temperature of 180°C were applied. Data were acquired in DIA mode utilizing a 3 × 8 dia-PASEF window scheme covering a mobility range of 0.64 to 1.45 V*s/cm^2^. A ramp time of 100 ms and advanced collision energy settings were used, resulting in a cycle time estimate of 0.95 s. For protein–cytosolic RNA crosslinking (cytosolic XRNAX), analysis via DDA occurred on an Orbitrap Astral mass spectrometer (Thermo Fisher Scientific), connected to an Evosep One LC system (Evosep). Tip-bound peptides were eluted using the 68 min (20 SPD) low-pressure gradient and separated on an Aurora Rapid XT analytical column (8 cm × 75 µm, IonOpticks) at a flow rate of 200 nl/min. The mass spectrometer was operated in positive ionization mode with a sprayer voltage of 2000 V. The full scans in the orbitrap (MS1) were acquired over a mass-to-charge (m/z) range of 360–1300 and the resolution was set to 240 000 at m/z 200. The maximum injection time (maxIT) was 10 ms at a normalized AGC target value of 300%. Peptides were isolated in the quadrupole using a 1.2 Th window, then fragmented with 26% HCD energy. For the MS2 scans in the Astral, the AGC target was set to 300%, maxIT to 3.5 ms, and the scan range to 100–2000 m/z. The LC settings for DIA were comparable to the ones used for DDA experiments. For DIA experiments, the mass spectrometer was operated in positive ionization mode with a sprayer voltage of 2000 V. The full scans in the orbitrap (MS1) were acquired over a mass-to-charge (m/z) range of 360–1000 and the resolution was set to 240 000 at m/z 200. The maximum injection time (maxIT) was 5 ms at a normalized AGC target value of 300%. For Astral DIA MS2 scans, the precursor range was set to 360–1000 m/z, with isolation windows of 4 Th. The collision energy for HCD was set to 28%, MS2 scan range was 150–2000 m/z, normalized AGC target was 300%, and maxIT was 3 ms. The DIA loops were time-controlled with precursor scans being scheduled in 0.6 s intervals. 

Protein–protein crosslinking samples were analyzed on an Orbitrap Astral mass spectrometer (Thermo Fisher Scientific) connected to a Vanquish Neo UHPLC system (Thermo Fisher Scientific). Peptides were resuspended in a solution containing 1.6% v/v acetonitrile and 0.1% v/v formic acid and were injected onto a 5.5 cm High Throughput µPAC™ Neo HPLC Column (Thermo Scientific) operating at 50°C column temperature. The mobile phase consisted of water with 0.1% v/v formic acid (mobile phase A) and 80% v/v acetonitrile with 0.1% v/v formic acid (mobile phase B). Peptides were eluted at a flow rate of 300 nl/min using a 50-min gradient: a linear increase of mobile phase B from 2% to 12.5% over 10 min, followed by an increase to 45% in 40 min, then to 55% in 2.5 min, and finally a ramp to 95% B in 2.5 min. Eluted peptides were ionized by an EASY-Spray source (Thermo Scientific) and introduced directly into the mass spectrometer. The mass spectrometry data was acquired using a DDA mode with the top-speed option. For each 2.5-s acquisition cycle, the full scan mass spectrum was recorded in the Orbitrap with a resolution of 120 000 with an m/z range of 400–1450. The AGC was set to 100% with a maximum injection time of 50 ms. Ions with charge of 3–7 and minimum intensity of 1E4 were then individually isolated with a 1.4 m/z isolation window and fragmented using higher-energy collisional dissociation (HCD) with a normalized collision energy of 30%. The fragmentation spectra were then recorded in the Astral mass analyzer. The AGC was set to 100% with a maximum injection time of 20 ms. Dynamic exclusion was enabled with single repeat count and 30 s exclusion duration.

### MS database search

For protein–RNA (XRNAX) and protein-DNA (XDNAX) crosslinking, MS data acquired in data-dependent (DDA) mode was searched with MaxQuant [30] (2.4.2.0). All searches were performed against the Uniprot human proteome (search term: “reviewed:yes AND proteome:up000005640,” 20216 entries, retrieved 13 June 2020). For searches and data processing outside of MaxQuant, its contaminants.fasta was included in the UniProt human proteome file for consistency with the flag “CON_.” MaxQuant settings were kept at their default value, except activation of the iBAQ quantification and setting peptide and protein identification FDR to 1 for PROSIT rescoring [31]. For the RNA-interacting proteomes responding to RNA-binding drugs, files were searched in three batches, each processed on the same day, with the “match between runs” option enabled. Search results were rescored using an in-house pipeline incorporating PROSIT and Picked Protein Group FDR [32]. For protein–RNA (XRNAX) and protein–DNA (XDNAX) crosslinking, MS data acquired in data-independent (DIA) mode was searched with DIA-NN [33] (1.9.1). The option “FASTA digest for library-free search/library generation” was selected with all default settings. For the analysis of RNA-interacting proteomes extracted after different irradiations (Fig. 3B), each file was searched individually to avoid the transfer of identifications. In the case of the RNA-interacting proteomes responding to RNA-binding drugs, files were searched in three batches analogously to the MaxQuant searches and with activated “MBR” to allow for transfer of identifications within one experiment. For protein–cytosolic RNA crosslinking (cytosolic XRNAX), all data was searched with MSFragger (4.3) within FragPipe (23.1). [34, 35] For MS data acquired in data-independent (DIA) mode the workflow “DIA_SpecLib_Quant” was loaded. For MSBooster, the Koina server was used to find the best models. [36] For MS data acquired in data-dependent (DDA) mode, the workflow “Basic-Search” was loaded, and phosphorylation (STY 79.96633) and ubiquitination (K 114.04293) were added as variable modifications in the MSFragger. For MSBooster, the Koina server was used to find the best models [36]. PTM-Shepherd defaults were loaded for offset search and quantification occurred with DIA-NN in FragPipe. For protein–protein crosslinking, MS raw data were converted to peak lists using the MSConvert module in ProteoWizard (version 3.0.11729). Precursor and fragment m/z values were recalibrated. Identification of crosslinked peptides was carried out using xiSEARCH software (https://www.rappsilberlab.org/software/xisearch, version 1.7.6.4) [37]. The peak lists were searched against sequences of the expressed AIFM1. The reversed protein sequence of the protein subunits was used as a decoy during the search for error estimation. The following parameters were applied for the database search: MS accuracy, 3 ppm; MS2 accuracy, 5 ppm; enzyme, trypsin (with full tryptic specificity); allowed number of missed cleavages, 2; missing monoisotopic peak, 2. For all samples, carbamidomethylation on cysteine was set as a fixed modification, and oxidation on methionine was set as a variable modification. Match to non-covalent links was enabled. The crosslinker was set to SDA. The reaction specificity for SDA was assumed to be for lysine, serine, threonine, tyrosine, and protein N-termini on the NHS ester end and any amino acids for the diazirine end. SDA loop link and hydrolyzed SDA on the diazirine end were set as variable modifications. Crosslinked peptide candidates with a minimum of three matched fragment ions (with at least two containing a crosslinked residue) in each crosslinked peptide were filtered using xiFDR (version 2.2.betaB) [38, 39] with the filter setting [scoreP1Coverage ≥ 3 AND (pepSeq2 is null OR scoreP2Coverage ≥ 3)] AND (site1 > 0 OR pepSeq2 is null) and ([%fragment unique crosslinked matched conservative%] ≥ 2.0). A false discovery rate of 5% at the residue-pair level was applied. xiVIEW [40] was used to visualize the crosslinking data on the crystal structure of AIFM1 (PDB 4BUR) and to measure the Euclidean distances between Cα atoms of the crosslinked residue pairs.

### Processing of proteomic data

All data analysis was performed in R (4.4.0) via RStudio (2024.04.2, build 764). For the analysis of proteomic data searched with MaxQuant (DDA acquisition) the proteinGroups_fdr0.01.txt file was used. Contaminants (“CON_”) and reverse identifications (“REV_”) were removed. In the case of data searched with DIA-NN (DIA acquisition), the report.pg_matrix.tsv as well as the report.pr_matrix.tsv file were used, and contaminants (“CON_”) removed. This was also true for data searched by MSFragger but quantified via DIA-NN in FragPipe. For the comparison of DNA-interacting proteomes between three cell lines, proteins detected with fewer than two peptides were removed, and iBAQ values were median-centered across all replicates. Missing values were imputed as described before [18] with an adaptation of the function described for PERSEUS [41], using the parameters width 0.3 and downshift 1.8. Negative binomial model-based (NBM) testing occurred with DESeq2 [42] under the assumption that iBAQ represents protein copy numbers on DNA [43]. Triplicates of each cell line were tested against triplicates of the mean. Fold changes were corrected using the apeglm package. [44]. To determine half-effective crosslinking times (ET_50_), DIA-NN LFQ values were first median-centered using the intensities of glycoproteins (retrieved from UniProt via “CARBOHYD”), which form a constant background of XRNAX, XDNAX, and other TRIZOL-based proteomics methods such as OOPS [10], that is enriched independent of crosslinking. LFQ values were normalized to the maximum and proteins with relative intensity >0.5 in the unirradiated sample removed as background. Time points before and including the maximum were used for fitting a linear model using log-transformed irradiation times. Slopes of the fit were used to calculate half-effective crosslinking times using the formula ET_50 _= log(2)/slope. For comparisons to the full proteome, we used a deep MCF7 total proteome that we recently reported [18]. RNA-interacting proteomes (XRNAX) were processed and analyzed in four batches, each comprising triplicates of four drug treatments and a DMSO control. Each batch included its own triplicate DMSO-treated controls, which were used as the reference for differential quantification within that batch. Differential testing was performed separately for each batch using DESeq2 with fold-change correction as described earlier. For DDA analyses, iBAQ values were used, and missing values were imputed as described earlier. Proteins detected with fewer than three peptides across all samples were excluded (Supplementary Table S4). For DIA analyses, DIA-NN LFQ values were used, and only proteins supported by more than two precursors were included in differential testing (Supplementary Table S3). Specifically, the report.pr_matrix.tsv file was used to exclude proteins supported by only a single precursor across all replicates. This substantially improved differential analyses by removing extreme and spurious results. Cytosolic RNA-interacting proteomes (cytosolic XRNAX) were analyzed using the same workflow, except that limma [45] was used instead of DESeq2 for differential testing. For DIA analyses, DIA-NN output files generated by FragPipe were filtered to remove single-fragment identifications as described earlier. For absolute quantification, LFQ values were converted to normalized iBAQ values as described elsewhere [46]. For DDA analyses, proteins with a maximum spectral count of one across all replicates were excluded, and MaxLFQ values were used for differential testing.

### Functional annotation of proteins and data visualization

Proteins were annotated via ENSEMBL BioMart accessed via the R package “biomaRt.” GO enrichment analysis was performed using the GOrilla web interface. Data were plotted using the R packages “ggplot2” and “ggbeeswarm.” Renderings of the UVEN device were performed in Fusion 360 (Autodesk). Figures were partially generated with Biorender. Figures were composed in Adobe Illustrator 2025 (Adobe).

## Results

### High-intensity UV irradiation accelerates photoreactions by orders of magnitude

In order to increase protein–DNA crosslinking yields with the poorly photoreactive nucleotide 4-thiothymidine, we recently reported “UV irradiation system for ENhanced photo-activation” (UVEN)—an LED-based irradiation device for high-intensity UV irradiation of culture cells or other biological specimens. [29] UVEN aims to improve the main shortcomings of a standard bulb-based device, which relate to low intensity of the light source and only ice as protection from heat and desiccation (Supplementary Fig. S1A). To increase intensity but limit toxicity of UV light toward living cells, the device uses longwave UV with 365 nm wavelength, avoiding photodamage of protein and nucleic acids absorbing light at 254–280 nm. [18] The employed high-powered UV-LEDs emit >2000 times stronger UV light than conventional bulbs (optically directly measured at the light source). In order to irradiate the area of a cell culture dish, sixteen LEDs are mounted on a large aluminum heat sink that is suspended in the irradiation chamber (Supplementary Fig. S1B and C). This chamber is isolated by a quartz glass window from the specimen chamber on top, which physically separates light source and biological sample into two chambers that can be cooled independently. This allows for very intense irradiation at controlled temperature (Supplementary Fig. S1D). During uniform illumination (∼2 W/cm^2^), UVEN is able to accelerate photoreactions homogeneously across an entire 15 cm cell culture dish by several orders of magnitude compared to conventional UV bulb irradiation. Figure 1B illustrates this on the example of a kinase inhibitor probe for PAL. LC-MS quantification demonstrated that UV bulb irradiation required >250 s, whereas UVEN irradiation required only 0.5 s for photoconversion of >90% of the initial compound, thus accelerating the photo-activation of the diazirine >500-fold. To irradiate smaller specimens such as tissue sections, it is possible to move the LEDs closer to the glass bottom, irradiating small areas (16 times 1 cm^2^) with intensities as high as 10 (±0.1) W/cm^2^, 1400 times more intense than a bulb-based device. We found that mouse tissues exhibited surprisingly high 365 nm light penetration under this configuration. When a 0.5 mm brain tissue section was irradiated at 10 W/cm², a residual light intensity of 1 W/cm² was measured (Supplementary Fig. S1E), which is >100-fold higher than the surface intensity of an unobstructed 365 nm UV bulb (∼7 mW/cm²). This implies that high-intensity 365 nm light could effectively activate photoreactions throughout brain tissue sections thicker than one millimeter. To make high-intensity photo-activation available to a wide community of researchers, we established a website that collects open-source construction plans, assembly manuals, software, and future versions of the device at www.uven.org.

### Sub-second protein-drug crosslinking in living cells

We tested high-intensity UV photo-activation on four proteomic applications, starting with in-cell protein-drug crosslinking. A commonly applied chemoproteomic approach to characterize drugs is PAL, which has been widely explored for drug-target deconvolution and fragment-based screens. [23, 47, 48] For PAL, a parent compound is modified with a photoreactive group and a click handle (Fig. 1B). The probe binds target proteins in living cells or lysates before UV activation covalently crosslinks them in a photoradical reaction, allowing for enrichment under highly denaturing conditions. Despite its utility in living cells, PAL often suffers from low crosslinking yields, even with prolonged irradiation of 10–20 min. [49] We used N-hydroxysuccinimide (NHS) chemistry to functionalize the CDK4/6 kinase inhibitor palbociclib in a single-step reaction without cleanup to a PAL probe (Supplementary Fig. S1F) [49], which we applied to living HeLa cells or native HeLa lysates. After irradiation-induced crosslinking, click chemistry was used to add a fluorescent dye, and photo-crosslinked protein-drug complexes were resolved by SDS–PAGE (Supplementary Fig. S1G). Strikingly, high-intensity UV-LED irradiation of both lysates and living cells led to rapid fluorescent labeling of specific proteins after only 0.5 s of photo-activation, while comparable labeling only occurred after minutes of irradiation with conventional UV bulbs (Fig. 1C and D). Photoligation in lysates was less specific than in intact cells, where primarily bands corresponding to the palbociclib targets CDK4/6 became apparent. In living cells, the labeling intensity after 1 s of high-intensity UV-LED irradiation was similar to the one achieved after UV bulb irradiation for 500 s, which is close to the commonly applied ∼10 min of irradiation used throughout the current literature [23, 49]. The background signal increased markedly after 1000 s of bulb or 10 s of LED irradiation, indicating that there is a trade-off between crosslinking yield and specificity. Overall, high-intensity UV-LED irradiation accelerated protein-drug photo-crosslinking 500-fold, for the first time enabling PAL in living cells under physiological culture conditions.

### High-yield protein–protein crosslinking for structural proteomics

We next turned toward protein–protein crosslinking. In recent years, the use of photo-activatable diazirine groups in crosslinking MS has gained popularity, as it not only increases crosslinking density [50] but also enhances the contrast and resolution of crosslinking data. [51] We first tested the performance of the UVEN irradiation device on diazirine-based protein photo-crosslinking *in vitro*. The isolated human protein AIFM1 forms a dimer in the presence of NADH. [24] We crosslinked the induced AIFM1 dimer using the photo-crosslinker sulfo-NHS-diazirine (sulfo-SDA). SDS–PAGE analysis revealed that a detectable crosslink-stabilized AIFM1 dimer was yielded with as little as 2 s of activation. The yield of the crosslinked dimer increased with longer UV exposure and plateaued at 32 s (Fig. 1E). According to previous studies [52, 53] and the manufacturer’s instructions, achieving an optimal sulfo-SDA activation typically requires ~20–30 min using conventional UV-bulb setups. When subjected to LC-MS/MS, the AIFM1 sample crosslinked with 32 s of UV activation yielded a total of 122 identified crosslinks (at 5% FDR at the linkage level), consistent with expectations for such an experimental setup without any enrichment for crosslinked peptides. [24] Ninety-nine of them could be mapped onto the crystal structure of the AIFM1 dimer (PDB 4BUR), with 86 (87%) having Cα–Cα distances below the calculated crosslinking limit of 30 Å (Fig. 1F). This high level of agreement with the crystal structure suggests that the activation did not cause evident structural perturbations. We next explored the potential of high-intensity LED irradiation for protein crosslinking in intact cells. Cultured HEK293 cells incorporating photo-L-leucine [28] were exposed to UV twice, each for 10 s. The reaction was monitored via western blotting of a biotin-tagged protein. As shown in Supplementary Fig. S1H, after UV exposure, the biotin-tagged protein appeared as a single band in the control sample of cells that had grown on regular L-leucine, whereas higher molecular weight bands emerged in the samples of cells that had grown on photo-L-leucine, indicating successful protein–protein crosslinking. In contrast, the same experiment using a UV bulb setup required 12 min of exposure. Additionally, the UVEN device irradiates from below, allowing crosslinking to proceed in the presence of culture medium rather than PBS. This minimizes cellular stress and enables a more physiologically relevant *in vivo* crosslinking workflow. In summary, UVEN effectively activated diazirine-mediated protein–protein crosslinking both *in vitro* and in intact cells. This significantly enhanced the temporal resolution of crosslinking MS experiments, particularly those aiming to monitor dynamic changes in protein structure and interactions over time.

### Photo-crosslinked DNA interactomes quantify genomic regulation across cell types

**Figure 2.**
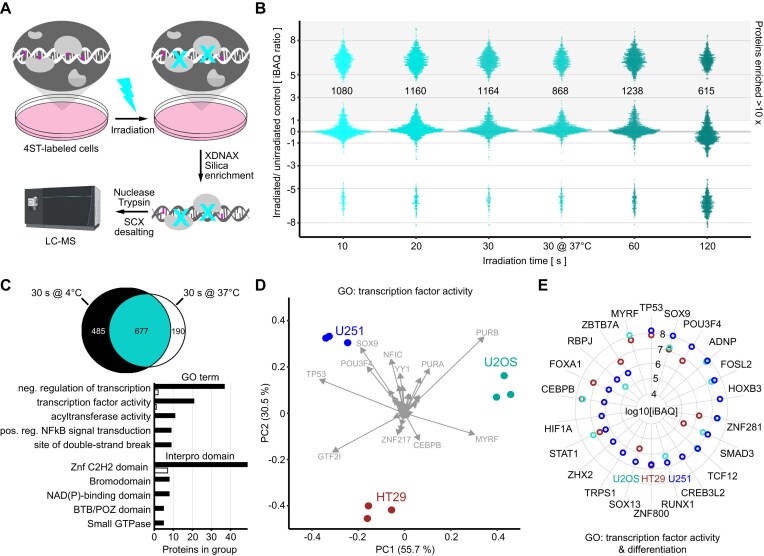
Quantitative comparison of DNA-interacting proteomes from different cell types. (**A**) Workflow for the proteomic analysis of protein–DNA interactions using photo-crosslinking. Cells are metabolically labeled with the photo-activatable nucleotide 4-thiothymidine (4ST), before high-intensity UV irradiation and purification of photo-crosslinked protein–DNA complexes for analysis by LC-MS (XDNAX). (**B**) Bee plot of protein abundances in MCF7 DNA interactomes with increasing UVEN irradiation time relative to unirradiated control cells. All irradiations occurred on cells in ice-cold PBS except for one on cells in their original medium straight from the incubator (30 s at 37°C). To display proteins without intensity in unirradiated cells pseudocounts were added to iBAQs. (**C**) Venn diagram comparing DNA interactomes of cells UVEN irradiated for 30 s in ice-cold PBS (4°C) or their original medium (37°C). Compared are proteins with >10-fold enrichment over unirradiated cells [see panel (B)]. Barplots show the top 5 GO terms and InterPro domains enriched in DNA interactomes derived at 4°C over 37°C. (**D**) Biplot for PCA of transcription factor abundances in DNA interactome of three cell lines originating from different germ layers. Replicates of each cell line are shown in same color. (**E**) Radar plot comparing abundances of transcription factors linked to differentiation in DNA interactome from three cell lines. Figure 2 was partly created in BioRender. Trendel, J. (2026) https://BioRender.com/6mlk4ok.

We next turned to protein–DNA crosslinking, where photo-crosslinking has remained largely unexplored due to various technical challenges. We have recently shown that metabolic DNA labeling with the photo-activatable nucleotide 4-thiothymidine (4ST), combined with high-intensity UV irradiation, strongly enhances the crosslinking of DNA-interacting proteins, enabling comprehensive analysis of the DNA-interacting proteome (Fig. 2A) [9]. To this end, photo-crosslinked protein–DNA complexes can be purified for LC-MS analysis using DNA as an affinity handle in a process we termed protein-crosslinked DNA extraction (XDNAX) (Supplementary Fig. S2A). To better characterize protein–DNA crosslinking kinetics with high-intensity UV, we recorded an irradiation time course in 4ST-photosensitized MCF7 cells. While conventional UV bulb irradiation failed to produce effective protein–DNA crosslinking, high-intensity LED photo-activation as short as 10 s enriched over 1000 proteins relative to unirradiated controls, 60% of which were Gene Ontology (GO) annotated as nucleic acid-binding (Fig. 2B). Extending irradiation beyond 30 s yielded only a marginal increase in DNA-crosslinked proteins (+6.4%), whereas irradiation longer than 60 s caused a marked decrease (−47%), indicating the onset of photodamage. Additionally, 30 s irradiations at 37°C in cell culture medium resulted in a 36% drop in the number of enriched proteins compared to 4°C in PBS (Fig. 2B), even though the overall abundance of proteins detected under both conditions remained consistent (R² = 0.93, Supplementary Fig. S2B). Comparison of GO annotations for proteins uniquely detected at each temperature revealed a notable enrichment of transcription factors and chromatin regulators, particularly C2H2-type zinc finger proteins, in DNA interactomes crosslinked at 4°C (Fig. 2C), as will be discussed further below. A comparison of MCF7 DNA interactomes to nuclear proteomes indicated that the DNA repair proteins MPG and MGMT were particularly abundant upon photocrosslinking, indicating that 4ST or its derivatives might be partially removed via the base excision repair pathway or by direct MGMT conversion. [54, 55]. To evaluate protein–DNA crosslinking across different cell types, we recorded DNA interactomes from three commonly used cell lines derived from different embryonic germ layers (U251 glioblastoma for ectoderm, HT29 colon adenocarcinoma for endoderm, U2OS osteosarcoma for mesoderm) and compared protein abundances in their DNA interactomes by label-free quantification (Supplementary Table S1). This differential comparison included 1147 proteins with a previous GO annotation as DNA or RNA binding, 118 of which have been previously reported as human transcription factors. [56] Principle component analysis (PCA) of transcription factor abundances clearly separated the three cell lines and highlighted individual transcription factors contributing most strongly to the separation (Fig. 2D). Most variance was explained by GTF2I, MYRF, PURB, SOX9, and TP53, which was undetected on DNA in U2OS cells but among the most abundant transcription factors on DNA in HT29 or U251 cells (Supplementary Fig. S2C and D). Notably, both cell lines are homozygous for the dysfunctional TP53 variant Arg273His associated with Li-Fraumeni syndrome, whereas U2OS carries a wild-type form of TP53. [57] The Arg273His mutation occurs at a DNA-contact site within a mutation hotspot of the TP53 DNA-binding domain and has been reported to change its binding specificity to orchestrate an oncogenic transcriptional program that drives cancer progression. [58] Overall, 45 transcription factors showed significant abundance differences between the three DNA interactomes, ten of which were exclusively found in the DNA interactome of U251 glioblastoma cells, including HOXB3, CREB3L2, and HIF1A [adj. *P* < .001, negative binomial model (NBM) testing toward mean, Supplementary Fig. S2E]. Indeed, independent genome-wide screens in mice and humans have identified HOXB3 and CREB3L2 as drivers of glioblastoma [59, 60], a cancer entity where overactivation of HIF1A often drives severe hypervascularization. [61] We observed distinct abundances of DNA for transcription factors with involvement in cell differentiation (Fig. 2E), and, specifically in U2OS cells, strongly decreased DNA association of various basal transcription factors involved in RNA polymerase II transcription regulation (Supplementary Fig. S2F). Overall, this comparison illustrates how photo-crosslinked DNA interactomes provide insights into genomic regulation across cell types, revealing quantitative fingerprints of transcription factor activities in direct contact with DNA.

### Rapid photo-crosslinking for the quantification of RNA-interacting proteomes under physiological culture conditions

**Figure 3.**
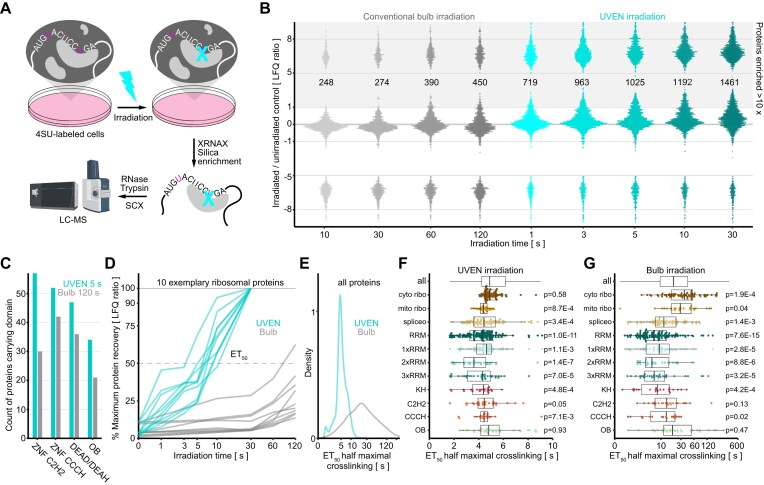
Quantitative analysis of RNA-interacting proteomes and their photocrosslinking kinetics. (**A**) Workflow for the proteomic analysis of protein–RNA interactions using photo-crosslinking. Cells are metabolically labeled with the photo-activatable nucleotide 4SU, before high-intensity UV irradiation in the original medium and purification of photo-crosslinked protein–RNA complexes for analysis by LC-MS (XRNAX). (**B**) Bee plot of protein abundances in RNA-interacting proteomes extracted after increasing irradiation time with a conventional bulb-based device or UVEN. An equivalent of 10 million MCF7 cells was analyzed by DIA on a timsTOF HT using a 22-min gradient; see Supplementary Fig. S3B for DDA comparison. To display proteins without intensity in unirradiated cells pseudocounts were added to LFQ values. (**C**) Barplot comparing occurrences of proteins with specific DNA-binding domains between RNA interactomes derived with 120 s bulb or 5 s UVEN irradiation. (**D**) Line plot comparing the recovery of 10 exemplary cytosolic ribosomal proteins after increasing crosslinking time and XRNAX. Each line represents one protein. Normalization occurred toward the maximum LFQ for each protein. (**E**) Density plots for half-maximal crosslinking times under UVEN or conventional bulb irradiation. Compared are all proteins (left panel) or proteins annotated in KEGG as part of the cytosolic ribosome, mitoribosome, and spliceosome (right panels). (**F**) Boxplots showing half-maximal crosslinking times between different protein groups under UVEN irradiation. Displayed are all proteins annotated in KEGG as part of the cytosolic ribosome (cyto ribo), mitoribosome (mito ribo), spliceosome (spliceo), proteins carrying RRM domains (RRM), of those carrying one, two, or three RRM domains (1×, 2×, 3× RRM), and K homology (KH), C2H2-type zinc-finger, and OB-fold (OB) domains. Testing occurred between all recovered proteins (all) and each group using a Wilcoxon rank-sum test and Bonferroni–Holm correction. Note linear scaling. (**G**) Same as in F but for conventional bulb irradiation. Note logarithmic scaling. Figure 3 was partly created in BioRender. Trendel, J. (2026) https://BioRender.com/6mlk4ok.

Finally, we turned to protein–RNA interactions, where various cellular processes, such as transcription [21], splicing [62], ribosome biogenesis [63], or translation [64], occur on a timescale of seconds to minutes. Conventional UV crosslinking with bulbs is therefore typically performed on ice to “freeze” molecular processes during the 1–2 min of irradiation (Supplementary Fig. S1A). We reasoned that faster crosslinking might eliminate the need to “freeze” processes, instead enabling snapshots of protein–RNA interactions under physiological culture conditions, with cells remaining in their original medium. To test this, we combined metabolic RNA labeling of expanding cells with the photo-activatable ribonucleotide 4SU and high-intensity photo-activation. [6] Moreover, we updated our previously published protocol for the extraction of protein-crosslinked RNA (XRNAX) [9] to analyze photo-crosslinked RNA interactomes via standard label-free proteomics, omitting trypsin predigestion but instead purifying intact protein photo-crosslinked to RNA before standard trypsin digestion (Supplementary Fig. S3A, see the “Materials and methods” section for details). This simplified the LC-MS analysis by eliminating the need for SILAC, allowing for conventional protein-level quantification by standard DDA and DIA methodology. We compared protein–RNA crosslinking with high-intensity UV-LEDs to conventional UV bulbs and irradiated 4SU-labeled cells straight from the incubator in their original medium for increasing durations (Fig. 3A). Strikingly, a single second of high-intensity LED irradiation led to enrichment of more proteins than 120 s of conventional UV bulb irradiation (Fig. 3B). Five seconds of LED irradiation more than doubled the number of enriched proteins compared to 120 s of UV bulbs, whereas longer irradiation times only marginally improved the outcome. Comparing identical samples analyzed via DIA with 22 min active gradient time on a timsTOF HT (Fig. 3B) to standard DDA analysis with 60 min active gradient on an Orbitrap Eclipse (Supplementary Fig. S3B), we found that RNA-interacting proteomes were highly amenable to DIA analysis, doubling the number of enriched proteins detected in the crosslinked samples. GO enrichment analysis indicated that this particularly included lower-abundant RNA-binding proteins involved in chromatin organization and mitochondrial translation (Supplementary Table S2). In summary, high-intensity LED irradiation dramatically accelerated protein–RNA crosslinking to just a few seconds, allowing for their fixation under physiological culture conditions. In combination with high-sensitivity DIA analysis this enabled quantification of near-complete RNA-interacting proteomes within 30 min of analysis time. 

### Quantitative and qualitative differences between rapid and slow protein–RNA photo-crosslinking

Next, we asked whether there were differences in the RNA-interacting proteomes derived by conventional bulb or high-intensity LED photo-activation. The absolute protein yields recovered by XRNAX were very similar for common proteins detected both after 120 s of bulb irradiation and 5 s of LED irradiation (Supplementary Fig. S3C and D). However, LED-irradiated samples were enriched with many additional proteins not detected at all in bulb-irradiated samples (Supplementary Fig. S3E). This suggested a qualitative advantage in crosslinking with high-intensity UV irradiation. Proteins uniquely enriched after 5 s of high-intensity irradiation were markedly lower in abundance within the RNA-interacting proteome compared to those also identified after 120 s of bulb irradiation (Supplementary Fig. S3F). We compared the abundance distributions of both groups in the MCF7 total proteome and found them to be very similar (Supplementary Fig. S3G), suggesting that the additional crosslinking was unlikely related to protein concentration in the cell. Interaction network analysis via STRING verified that proteins only identified by UVEN were strongly enriched in RNA-binding proteins (FDR = 1.1E−78), with the largest interaction clusters relating to mitochondrial translation, translation initiation, and messenger RNA (mRNA) splicing. The most common domain among them was the C2H2-type zinc finger domain, which occurred only half as often after UV bulb irradiation (Fig. 3C). Notably, under high-intensity LED irradiation C2H2-type zinc-finger proteins were among the proteins with the best recovery of all proteins in the RNA-interacting proteome relative to the total proteome (Supplementary Fig. S3H). To assess the crosslinking behavior of all proteins enriched by XRNAX, we compared absolute yields of purified proteins across different irradiation time points using label-free quantification. In most cases, we observed logistic growth for the amount of RNA-crosslinked protein with increasing irradiation time, allowing us to determine half-effective crosslinking times (ET_50_, Supplementary Table S2; see the “Materials and methods” section for details) (Fig. 3D). As expected, ET_50_ values were on average substantially shorter under LED irradiation and spanned a much narrower time window (Fig. 3E). This was particularly prominent for ribosomal proteins, which displayed a broad range of ET_50_ values under bulb irradiation but a remarkably narrow distribution under UVEN irradiation (ET_50_ standard deviation σ = 0.7 s versus 135.7 s; Fig. 3F and G). We used ET_50_ values to compare different RNA-binding domains and found particularly short crosslinking times for proteins containing RRM (adjusted *P* = 1E−10, Wilcoxon rank-sum test with Bonferroni-Holm correction) or KH domains (adjusted *P* = 9.4E−4). Proteins carrying two RRM domains showed very rapid crosslinking (adjusted *P* = 7.1E−7), the fastest being the poly-U binders CPEB2 and CPEB4, which also exhibited the strongest relative recovery compared to the total proteome (Supplementary Fig. S3H). Importantly, we observed very low correlation between protein abundances in the MCF7 total proteome and ET_50_ values for both bulb (Pearson R² = 0.05) and LED (R² = 0.09) irradiation (Supplementary Fig. S3I). Furthermore, the abundance distributions in the MCF7 total proteome were virtually identical for proteins with short and long ET_50_ values (Supplementary Fig. S3J), again verifying that 4SU crosslinking occurred independent of cellular protein concentration. Overall, these findings showed that rapid protein-RNA crosslinking with high-intensity irradiation provided qualitative and quantitative advantages over slow crosslinking with low-intensity irradiation, which failed for many proteins to produce sufficient yield at physiological temperature.

### Differential quantification of RNA-interacting proteomes upon acute drug action

**Figure 4.**
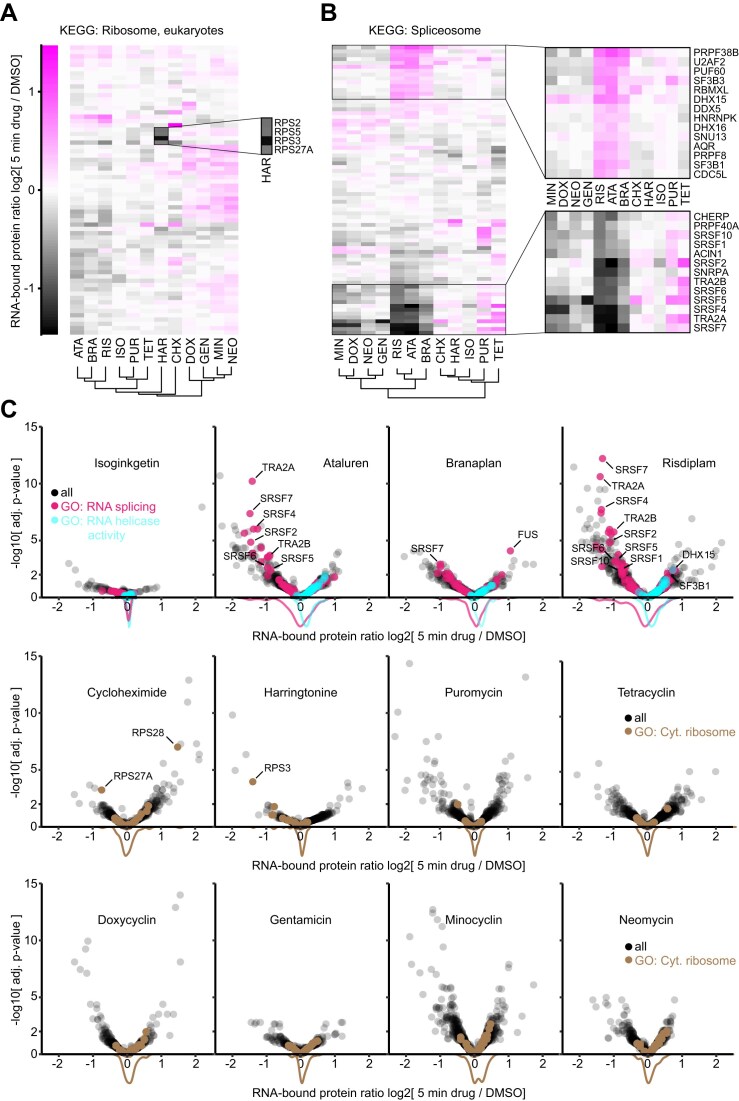
Quantitative comparison of RNA-interacting proteomes in response to RNA-active drugs. (**A, B**) Heatmaps comparing changes in RNA-interacting proteomes of MCF7 cells treated with 12 RNA-active drugs for 5 min compared to a mock-treated control (DMSO). ATA: ataluren, BRA: branaplan, RIS: risdiplam, ISO: isoginkgetin, PUR: puromycin, TET: tetracycline, HAR: harringtonine, CHX: cycloheximide, DOX: doxycycline, GEN: gentamicin, MIN: minocycline, NEO: neomycin. (A) Displayed are all detected constituents of the cytosolic ribosome. Inset magnifies proteins with strongest loss in RNA interaction. (B) Same as in panel (A) but for constituents of the spliceosome. Insets magnify proteins with particularly strong gain or loss in RNA interaction. (**C**) Volcano plots comparing the effect of 12 RNA-active drugs compared to a mock-treated control (DMSO) for all detected proteins in the RNA-interacting proteome. Density plots indicate fold change distributions of proteins involved in RNA splicing (magenta), RNA helicases (cyan), or the cytosolic ribosome (brown).

We benchmarked rapid protein–RNA crosslinking with high-intensity photo-activation on the quantitative analysis of RNA-interacting proteomes from MCF7 cells responding to a panel of 12 RNA-binding drugs. Since only seconds of irradiation were required and cells could remain in their original media during photo-crosslinking, we chose a short treatment time of 5 min to capture immediate changes in protein–RNA interactions during early drug engagement. For LC-MS analysis, we compared two single-shot approaches using the 22-min DIA method and the 60-min DDA method described earlier. DIA analysis enabled median differential quantification of 1728 proteins, and DDA 409 (Supplementary Fig. S4A and Supplementary Tables S3  and S4, adj. *P* < .01, NBM testing; see the “Materials and methods” section for details). On average, 20 proteins identified by DDA showed significantly altered RNA interactions, compared to 47 proteins with DIA, primarily increasing the quantification of lower-abundant proteins (Supplementary Fig. S4B). With a Pearson R^2 ^= 0.93, we observed good correlation in fold changes of RNA interaction for proteins identified as significantly regulated by both acquisition methods (adj. *P* < .01 in DIA and DDA), in line with a previous report that used conventional UV irradiation and a different extraction method (TRAPP). [65] RNA-interacting proteomes from cells treated with risdiplam showed one of the largest benefits from DIA analysis (Supplementary Fig. S4C), revealing differential RNA interaction for more than double the number of proteins than DDA. This included various proteins involved in RNA splicing, demonstrating that DIA provided additional, functionally relevant information on the activity of the splicing modulator (Supplementary Fig. S4C and D). As our drug panel contained compounds known to affect translation and splicing, we focused our analysis on protein constituents of the ribosome and spliceosome. Figure 4A summarizes all drug-induced changes in RNA interactions among constituents of the cytosolic ribosome, highlighting harringtonine, which strongly reduced RNA interactions for a selected set of small ribosomal subunit proteins. Particularly decreased interaction was observed for RPS3 (adj. *P* = 1.1E−4), whose contact with the mRNA at the entry channel is known to stabilize preinitiation complexes at the start codon. [66] This aligned with the known mode of action of harringtonine, which traps 80S ribosomes after the formation of the initial peptide bond, blocking the formation of consecutive preinitiation complexes. [64, 67] Turning to the spliceosome, we observed extensive remodeling of protein–RNA interactions for risdiplam and ataluren, as well as somewhat weaker responses for branaplan, minocycline, and doxycycline (Fig. 4B). Risdiplam and ataluren in particular elicited a strong effect on SR proteins, nine out of 13 of which significantly decreased their RNA interaction under risdiplam (adj. *P* < .01, Fig. 4C). A direct comparison of the two compounds indicated that both interfered in remarkably similar ways with proteins involved in exon definition and early splice site selection (SNRPA, SR proteins; Fig. 4B), while increasing RNA interactions of branch point interactors (U2AF2, PUF60, SF3B1, SF3B3), as well as helicases involved in later splicing stages (DHX16, DHX15, AQR). [68] We speculate that both compounds stabilized structures within the pre-mRNA substrate that required extensive helicase activity for splicing to continue, slowing down steps following 5′ splice site definition. A specific effect of risdiplam was strongly decreased RNA interaction of RNPS1 (adj. *P* = .002, Supplementary Fig. S4E), which has been shown to promote exon skipping [69, 70], potentially relating to the ability of risdiplam to promote exon inclusion. Overall, this confirmed that our optimized XRNAX workflow, combined with enhanced photo-crosslinking using the UVEN device, enabled the quantitative analysis of RNA-interacting proteomes with a temporal resolution of minutes under physiological culture conditions.

### Early cytosolic protein–RNA dynamics reveal upstream events of the UV-induced ribotoxic stress response

**Figure 5.**
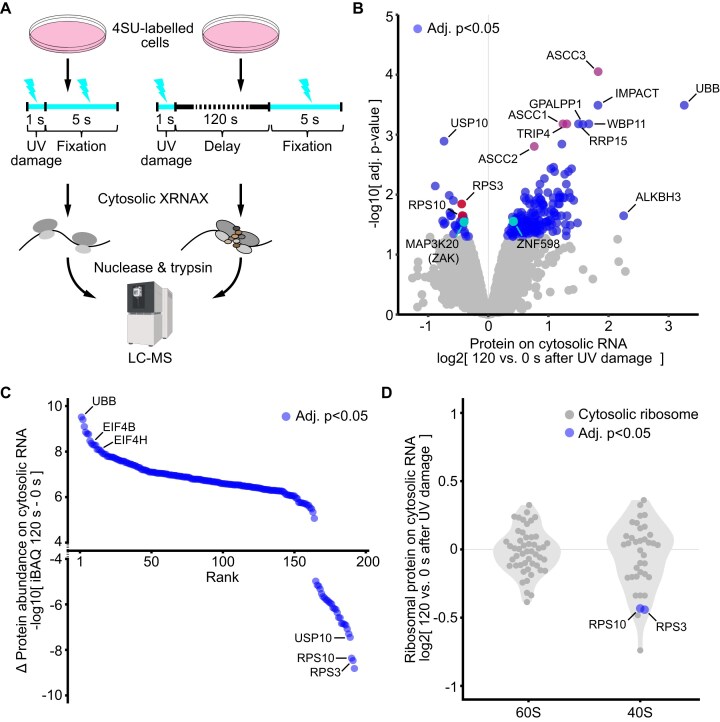
Cytosolic protein–RNA interactions minutes after UV damage. (**A**) Experimental scheme for the quantification of cytosolic protein–RNA interactions after UV damage. MCF7 cells with metabolically 4SU-labeled transcriptomes receive a 1 s UV pulse to induce damage, followed by a 5 s pulse to capture proteins present on RNA directly after or 120 s later. Cytosolic protein–crosslinked RNA is purified by nuclear extraction and silica enrichment (cytosolic XRNAX; see Supplementary Fig. S5A), and protein interactors are quantified by MS and a DIA method (see Supplementary Fig. S5 for DDA). (**B**) Volcano plot comparing cytosolic RNA interactomes immediately after UV damage to 120 s later. (**C**) Ranked scatter plot comparing changes in absolute protein abundances on RNA directly after UV damage to 120 s later. Only proteins with significant changes in panel (B) are shown (adj. *P* < .05). (**D**) Beeplots comparing fold changes from panel (B) only for constituents of the cytosolic ribosome. Figure 5 was partly created in BioRender. Trendel, J. (2026) https://BioRender.com/6mlk4ok.

Recently, it has been shown that damage to cytosolic RNA and ribosome stalling, rather than damage to nuclear DNA, is the primary driver of sunburn, i.e. UV-induced cell death of keratinocytes followed by inflammation and epidermal thickening. [71, 72] Mechanistically, ribosomes stalled by bulky UVA lesions or UVC-induced protein–mRNA crosslinks provoke ribosome collisions. [73, 74] Unresolved disomes activate the sensor kinase ZAK, which triggers the ribotoxic stress response by activating p38, leading to cell-cycle arrest, or JNK, leading to apoptosis. [71, 72, 75] So far, proteomic studies have used 30–60 min after UV damage as the earliest time points to capture the responding RNA interactome. [73, 74] As ZAK activation occurs already within 5 min after UV exposure, we sought to identify proteins involved in the very early RNA damage response. [72]. Exploiting the very rapid protein–RNA crosslinking enabled by our UVEN device, we devised an experimental strategy to quantify protein recruitment to cytosolic RNA as early as 2 min after UV damage. Because two minutes are typically required for protein–RNA photo-crosslinking using a conventional bulb-based irradiation setup, experiments on this timescale were previously impossible. We metabolically labeled the transcriptome of MCF7 cells with 4SU and applied a one-second UVEN irradiation pulse to introduce RNA photodamage in the form of protein–RNA crosslinks (Fig. 5A) [74]. Subsequently, a second irradiation was applied to capture protein interactors recruited to RNA either immediately or after a 2-min recovery period. To overpower the amount of protein crosslinked during the first pulse, this second pulse was five-fold longer, ensuring that the resulting photo-crosslinked RNA interactomes were dominated by proteins fixed during the second irradiation. Cytosolic protein–crosslinked RNA was then purified by hypotonic nuclear extraction followed by silica enrichment, and co-purified, crosslinked protein cleaned up for quantification by LC-MS (cytosolic XRNAX, Supplementary Fig. S5A; see the “Materials and methods” section). Strikingly, we observed highly significant recruitment of ASCC3 (Fig. 5B, adj. *P* < 8.9E−5, limma testing, Supplementary Table S5) comparing proteins associated with cytosolic RNA immediately after UV damage to those detected 120 s later. As part of the ASCC complex, ASCC3 has been shown to disassemble collided ribosomes through its ATP-dependent helicase activity, leading to splitting of the lead ribosome in a collision queue. [76] We also observed recruitment of all other members of the ASCC complex (ASCC2, ASCC3, TRIP4, adj. *P* < 1.6E−3), as well as strongly increased interaction of ALKBH3 (4.8-fold increase, adj. *P* = .02). ALKBH3 is a known ASCC3 interactor capable of removing base methylations from tRNA and mRNA, which are potent inducers of ribosome stalling. [77, 78, 76] Moreover, we detected significant recruitment of the ribosome collision sensor and E3 ligase ZNF598 (Fig. 5B, adj. *P* = .03) [76], accompanied by a strong increase in ubiquitin (UBB) levels (3.9-fold increase, adj. *P* = 2.1E−4). In fact, ubiquitin showed the largest increase in absolute protein copy numbers as estimated by iBAQ (Fig. 5C). Among the proteins exhibiting the largest absolute decreases were the small ribosomal subunit proteins RPS3 and RPS10, as well as the deubiquitinase USP10. Both RPS3 and RPS10 are central targets of ZNF598. Their ubiquitination initially promotes ASCC3 recruitment and, following subunit splitting, mark 40S subunits for lysosomal degradation, which can be antagonized by removal of ubiquitin by USP10. [76, 79] However, in our case USP10 appeared to dissociate from RNA, potentially amplifying ZNF598-mediated activity at early stages after damage. Interestingly, small and large ribosomal subunit proteins did overall not show significant changes in RNA interaction, except for RPS10 and RPS3 (Fig. 5D). We observed decreased RNA interaction of ZAK (Fig. 5B, MAP3K20; adj. *P* = .03, limma testing), which associates with ribosomes until collisions trigger its autophosphorylation and release to initiate ribotoxic stress signaling. [75] Although we did not detect the ribosome collision sensor GCN2 on cytosolic RNA, we observed highly significant recruitment of its inhibitor IMPACT (adj. *P* = 0.4E−4). This suggested that, at this early time point after damage, IMPACT suppressed activation of the integrated stress response and EIF2α phosphorylation to avoid premature, general translational arrest, while attempts to clear stalling ribosomes were still on their way. [80]. Analysis of the same samples by DDA instead of DIA (identical 50-min active gradient on an Orbitrap Astral) recapitulated most of these findings, albeit with reduced proteomic depth and statistical power (Supplementary Fig. S5B and Supplementary Table S5). Without further enrichment and using the DDA data, we searched for ubiquitin (Gly–Gly) modifications and detected increased ubiquitination at several sites on translation-associated proteins previously implicated in responses to formaldehyde-induced protein–RNA crosslinks (Supplementary Fig. S5C). [74, 81] Finally, in both DIA and DDA analyses, we observed especially significant recruitment of three proteins, WBP11, GPALPP1, and RRP15 (adj. *P* < 9.8E−3), which have not previously been linked to UV exposure or ribosome collisions and thus represent potential candidates for future studies on acute RNA damage. Overall, our analysis provides a unified view of the RNA-interacting proteome involved in the very early ribotoxic stress response, which until now has only been investigated at much later time points.

## Discussion

### Technical considerations and limitations of high-intensity UV crosslinking

Conventional bulb-based devices for the activation of photoreactions are limited by their photon output and their ability to protect biological samples during irradiation. One reported setup increased UV intensity by densely packing standard UV bulbs to illuminate an irradiation chamber from both the top and bottom. [82] This approach achieved an intensity of 0.2 W/cm²—approximately a 50-fold increase compared to conventional devices—likely representing the maximal achievable intensity with back-to-back packed UV bulbs without mirrors or lenses. Although lasers in the UVA range with very high intensities exist, their irradiation field is typically no larger than 1 mm, making it prohibitively costly to build an array capable of illuminating a cell culture dish with full intensity. Yet, individual UV lasers and UV LEDs have been used for photo-crosslinking in small vials. [2, 83–85] UVEN uses an array of UV-LEDs to irradiate an area of over 175 cm² with intensities of around 2 W/cm²—approximately a 500-fold increase compared to conventional bulb devices—without reaching the maximal LED packing density. Further acceleration of crosslinking reactions with more intense UV light could be desirable for in-cell kinetic studies. [2, 85] Employing additional LEDs, future versions of UVEN will increase the intensity by another order of magnitude, potentially pushing most photoreactions in cells to completion in fractions of a second. A current limitation of LED-based photoactivation is that the shortest readily available wavelength capable of delivering truly high-intensity light is 365 nm, whereas LEDs emitting at shorter wavelengths provide only a fraction of this power. In particular, activation of thiolated nucleotides, which absorb most efficiently around 330 nm, is suboptimal at 365 nm, and irradiation at shorter wavelengths would further accelerate crosslinking. The presented version of the device uses 16 LEDs, which can lead to variation in the received dose across cells of a culture dish. This shortcoming has been addressed in a new version (UVEN mk3), which now uses 100 LEDs that are more evenly spaced (www.uven.org). High-intensity UVA irradiation generates reactive oxygen species, which may affect experimental outcomes, especially during longer irradiations when cells remain exposed to the same medium for extended periods. We have also observed that red tissue slices, for example from muscle or liver, bleach readily during irradiation, presumably due to photodecomposition of flavins and porphyrins. We speculate that a substantial fraction of the variability in tissue penetration of 365 nm light observed in Supplementary Fig. S1E arises from differing levels of these chromophores across tissues. Nevertheless, we previously showed that even after prolonged exposure to high-intensity UV irradiation, cancer cell lines retain their ability to proliferate, implying that photodamage at 365 nm for cultured cells is limited. [29]

### Links between temperature and crosslinking efficiency of proteins with nucleic acids

During the analysis of DNA and RNA-interacting proteomes, we observed that for a group of proteins, crosslinking efficiency was sensitive either to temperature or UV intensity, often affecting zinc-finger proteins of the C2H2 type (Supplementary Fig. S4F). Proteins showing sensitivity in both DNA and RNA crosslinking contained between five and 15 C2H2 type domains per protein, suggesting that impaired crosslinking was not due to a low number of C2H2-type repeats. Recent studies using nucleotide-peptide hybrids to map DNA [18] or RNA [86, 87] interactions at site-level resolution reported very few crosslinks for zinc-finger proteins in their C2H2-type domains but primarily in flexible linkers between C2H2-type repeats or other intrinsically disordered regions. Interestingly, it has been reported that the flexible linkers between C2H2-type domains are critical for DNA binding, and their phosphorylation is a common mechanism for deactivation of C2H2-type zinc-finger proteins during mitosis. [88] Intrinsically disordered sequences typically interact with nucleic acids in a variety of conformational states [89], only some of which can lead to a successful crosslink. We speculate that in the case of C2H2-type zinc-finger proteins, this results in conformational gating of the crosslinking reaction at physiological temperature, when molecular motion is high and conformations change rapidly. Nevertheless, crosslinking can still be achieved by using a sufficiently reactive photoprobe—such as 4SU, but not 4ST—in combination with high photon flux during irradiation, as provided by high-intensity UV-LED but not by conventional UV bulbs. This illustrates how crosslinking of unstructured regions with low nucleic acid affinity can be affected by nearby high-affinity nucleic-acid binding domains, temperature, and light intensity.

### High-intensity photoactivation solves old problems and opens new avenues

We demonstrate that UVEN enhances photo-activation across various disciplines of photo-crosslinking, both *in vitro* and in living cells. UVEN accelerated in-cell photo-activation of a PAL probe from 10 min to just 1 s under physiological culture conditions. This not only implies a substantial gain in throughput but also improves sample handling in a way that enables previously inconceivable PAL-based drug and fragment screens. [23, 48, 49, 90] Similarly, high-intensity photo-activation reduced the time required for effective protein–RNA crosslinking to few seconds while cells remained in their original media. It has been reported that changes in temperature or extracellular osmolality are quickly countered by the cell via formation of molecular condensates that free up intracellular water, thereby avoiding cellular damage or dysfunction. [91] Hence, rapid crosslinking at physiological culture temperature at constant osmolality might prevent spurious interactions that only occur when cells are washed with cold saline or placed on ice. [92] Indeed, using rapid, high-intensity crosslinking, we observed highly consistent quantification of RNA interactomes from cells treated with RNA-binding drugs, demonstrating that minimizing perturbations during the crosslinking process significantly enhances the quantitative analysis of protein–RNA interactions. Finally, we show that substantially reduced crosslinking times combined with markedly improved crosslinking yields open new perspectives for studying cellular responses to RNA damage. We anticipate that this greatly enhanced temporal resolution will not only benefit investigations of ribosome collisions and the ribotoxic stress response, but will also reveal unprecedented detail on DNA damage or replication, transcription, splicing, and other processes that proceed on timescales of seconds to minutes.

## Funding

We gratefully acknowledge funding by the German Research Foundation DFG supporting this work [DFG project number 492625837 and project number 325871075 (SFB1309-B02)]. We also acknowledge funding from the Bavarian Research Foundation for construction of the UVEN irradiation system (BFS project number AZ-1444-20C).

## Supplementary Material

gkag339_Supplemental_Files

## Data Availability

Proteomic data and search results have been deposited in the MassIVE database under the identifier MSV000097903.
