# Supplementary material for: Rapid photo-crosslinking in living cells reveals protein–nucleic acid dynamics on a timescale of minutes
Source: Nucleic Acids Res. 2026 Apr 21;54(7):gkag339. doi: 10.1093/nar/gkag339 (PMC13096800; doi:10.1093/nar/gkag339)
Supplement: gkag339_Supplemental_Files [file gkag339_supplemental_files.zip › HighUV_Supplementary_figures_NAR_reviews1.docx]

**Supplementary Figures: Rapid photo-crosslinking in living cells reveals protein-nucleic acid dynamics on a timescale of minutes**

**
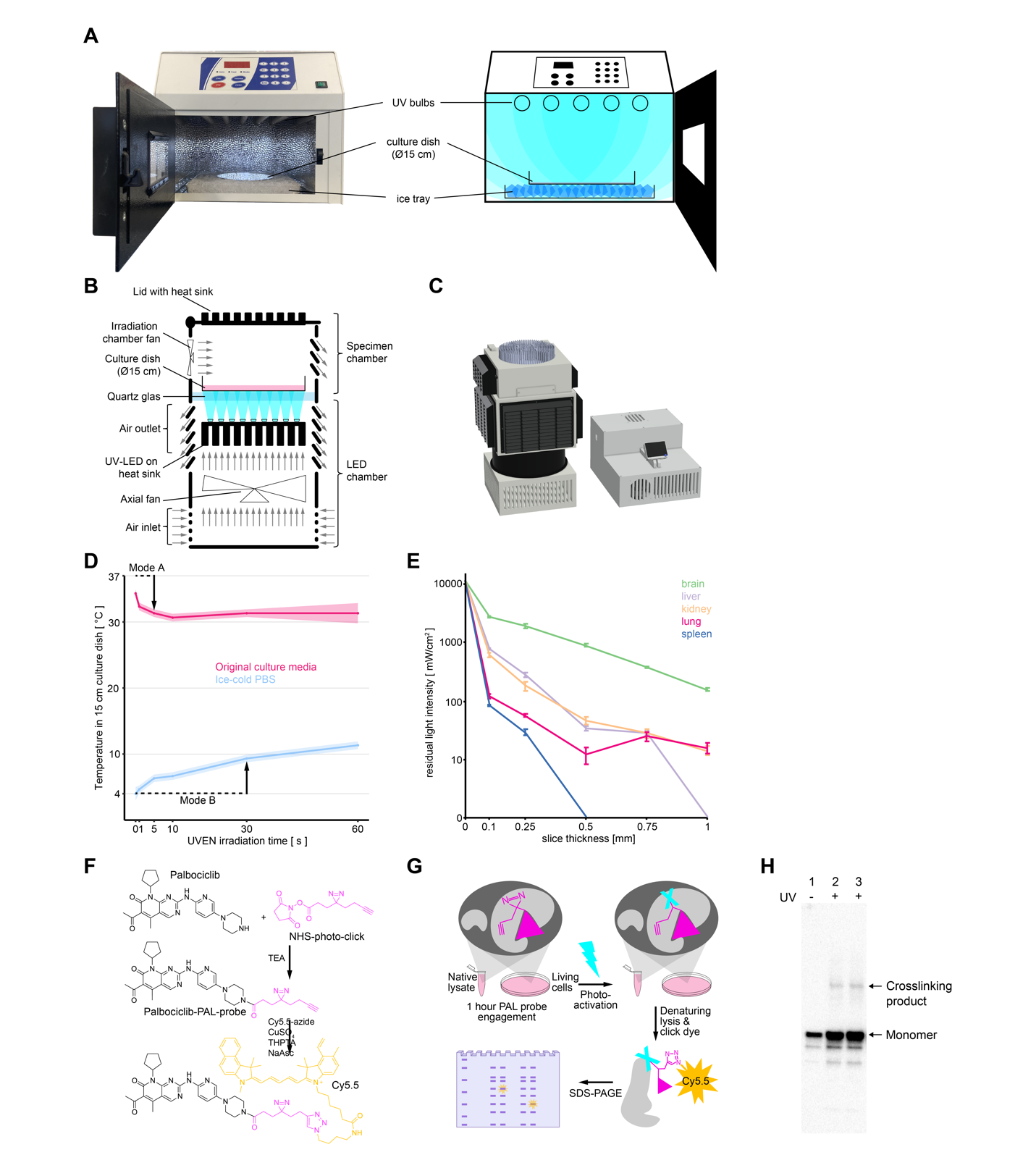
Figure S1: UV irradiation devices for the activation of photoreactions in biological specimens.
A)** Photo and schematic of a standard, bulb-based UV irradiation device. To irradiate adherent cultured cells, the medium is removed, and the culture dish is placed on an ice tray positioned beneath an array of UV bulbs.
B) Schematic representation of the UVEN irradiation device. High intensity UV-LEDs serve as irradiant, which illuminate a biological specimens through a glass window from below.
C) Rendering of the UVEN irradiation device and its control unit. The power supply and control electronics are located in a second housing (right) separate from the irradiation tower (left, see also B).
D) Line plot displaying the temperature development of 20 ml culture medium (red, 37 °C initially) or PBS (blue, 4 °C initially) in a 15 cm diameter culture dish during UVEN irradiation.
E) Line plot showing UV transmission from a high-intensity UV-LED through mouse tissue slices of increasing thickness, mounted between two glass slides. A light-tight blind was used to eliminate stray light, ensuring that only light passing through the tissue slice reached the UV meter (LS-128, Linshang).
F) Chemical structures for the reaction of the kinase inhibitor palbociclib to a PAL probe, and further derivatization with a fluorescent dye (Cy5.5) via copper-catalyzed click chemistry for in-gel imaging.
G) Fluorescence imaging of SDS-PAGE after PAL in native lysates. Compared are various time points of LED irradiation (UVEN) to conventional bulb irradiation at identical distance.
H) Western blot analysis of L-photo-leucine–mediated crosslinking in intact cells. The biotin-tagged protein appears as a single band under the control condition without L-photo-leucine incorporation (lane 1). Higher molecular weight crosslinked products are observed in the L-photo-leucine–incorporated samples (two replicates, lanes 2 and 3) upon 20 s of UV activation.
Figure S1 was partly created in BioRender. Trendel, J. (2026) https://BioRender.com/6mlk4ok

**Figure S2: Comparison of transcription factor activity via DNA-crosslinked proteomes from three cell lines.**A) Schematic for the extraction of protein-crosslinked DNA (XDNAX) via TRIZOL extraction and silica column purification.
B) Scatter plot comparing the abundances of proteins in DNA-interacting proteomes derived from cells crosslinked at 4 °C versus 37 °C.
C) Density plot showing the distribution of transcription factor abundances in DNA-interacting proteomes from three different cell lines. Mutant TP53 is abundantly detected on DNA in HT29 and U251 cells, while wild-type TP53 in U2OS cells remains undetected.
D) Bar plot comparing the abundances of ten transcription factors showing the most significant differences across the three cell lines. A negative binomial statistical model (NBM testing) was applied to each cell line individually, using the mean across all three as a reference (see Methods for details).
E) Heatmap illustrating the abundances of transcription factors with the most significant deviations from the mean (p < 0.001, NBM testing). Protein abundance in each triplicate was normalized to the mean abundance across all cell lines.
**F)** Radar plot comparing the abundances of basal transcription factors in the DNA-interacting proteomes of three cell lines.


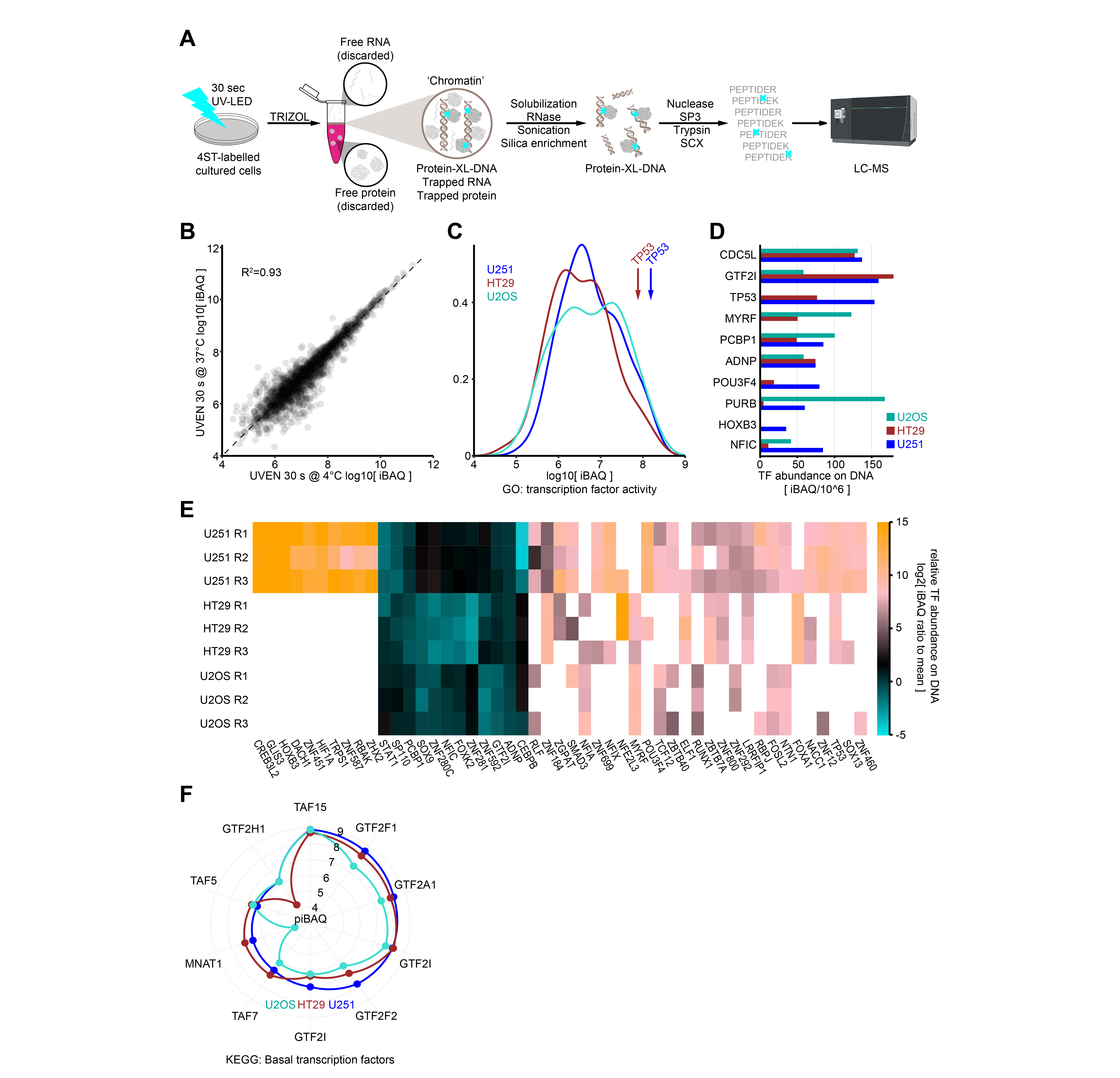


Figure S2 was partly created in BioRender. Trendel, J. (2026) https://BioRender.com/6mlk4ok

**Figure S3: Crosslinking kinetics of protein-RNA interactions using 4SU with low or high-intensity UV.**A) Schematic for the extraction of protein-crosslinked RNA (XRNAX) via TRIZOL extraction and silica column purification. Presented is an updated protocol (see Methods for details), that forgoes trypsin predigestion before the silica enrichment to allow for consistent label-free quantification of RNA-interactomes between conditions.
B) Beeplot of protein abundances in RNA-interacting proteomes extracted after increasing irradiation time with a conventional bulb-based device or the UVEN. An equivalent of 10 million MCF7 cells was analysed by DDA on an Orbitrap Eclipse using a 60-min gradient, see Figure 3B for DIA comparison. To display proteins without intensity in unirradiated cells pseudocounts were added to LFQ values.
C) Boxplots comparing protein abundances between RNA-interacting proteomes normalized to 5 s of UVEN irradiation.
D) Scatter plot showing protein abundances of proteins in RNA-interactomes derived after 120 s bulb or 5 sUVEN irradiation (see Figure 3B). Compared are only proteins enriched more than tenfold in both samples compared to an unirradiated control.
E) Venn diagram showing overlap between proteins enriched more than tenfold compared to an unirradiated control (see Figure 3B).
F) Histogram comparing protein abundances in RNA-interacting proteomes between groups in E.
G) Histogram comparing protein abundances in MCF7 total proteome between groups in E.
H) Beeplot showing recovery of proteins in RNA-interacting proteomes crosslinked for 5 s with UVEN relative to the MCF7 total proteome. Shown is the ratio of z-scored protein abundances, testing occurred with a Wilcoxon ranksum test between the indicated groups and all proteins.
I) Scatter plot comparing half-maximal crosslinking times (ET_50_) with protein abundances (iBAQ) in a deep MCF7 total proteome. Each point represents one protein; UVEN data is shown in cyan, bulb in grey.
J) Density plot showing protein abundances in a deep MCF7 total proteome. Compared are proteins with long or short UVEN ET_50_ values, as well as all proteins with a GO annotation for RNA binding.


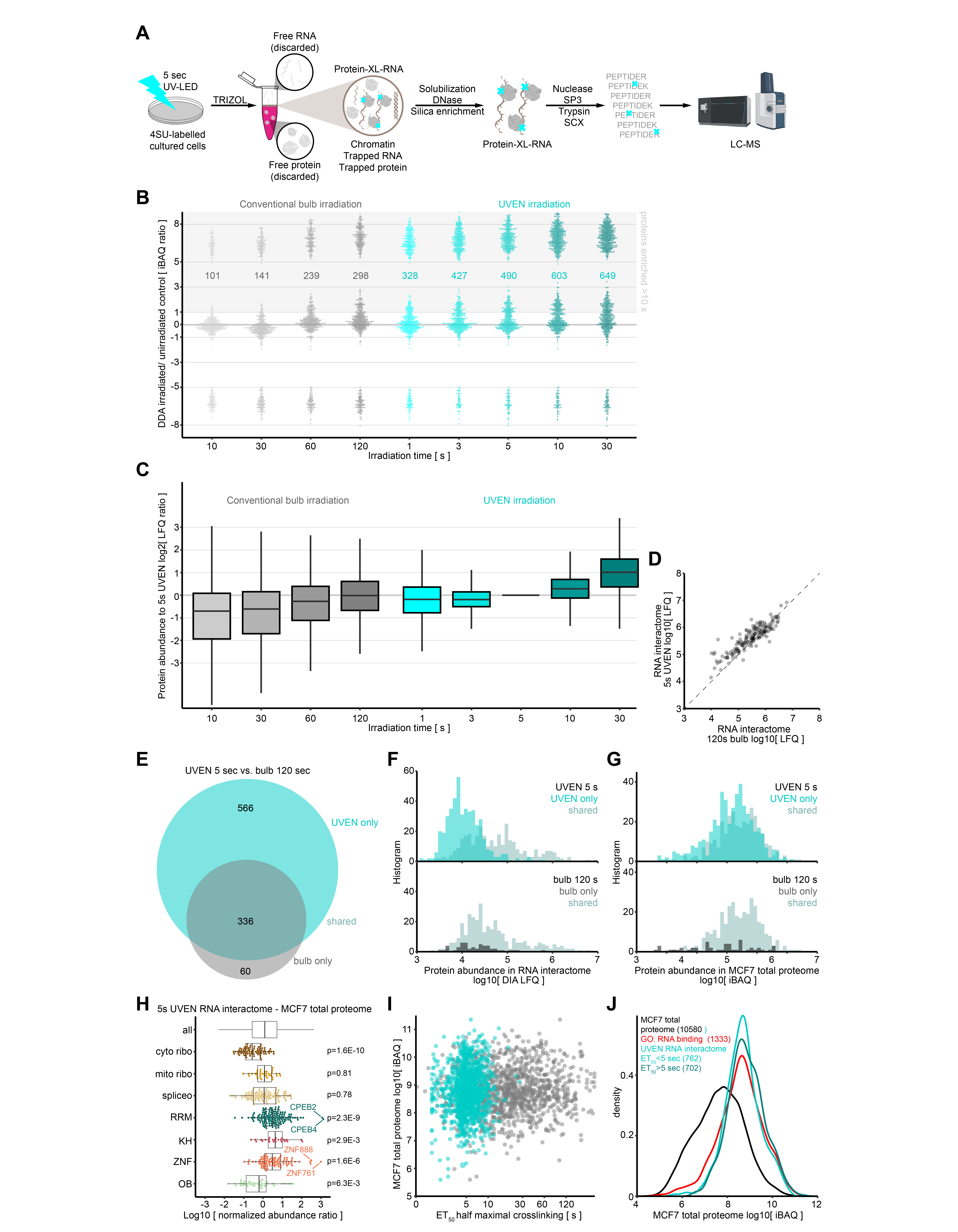


Figure S3 was partly created in BioRender. Trendel, J. (2026) https://BioRender.com/6mlk4ok

**Figure S4: Differential analysis of RNA-interacting proteomes via DDA and DIA methodology.**A) Barplots displaying proteins quantitatively compared in RNA-interacting proteomes from cells treated with different RNA-binding drugs. Identical samples were injected on different LC-MS systems for comparison, see Methods for details. Upper panel: All proteins in differential analysis towards mock-treated cells after imputation of missing values for triplicates. Lower panel: Proteins found with significant differences (adj. p<0.01 after NBM testing).
B) Comparison of proteins abundances of in RNA-interacting proteomes detected via DDA or DIA analysis. Top left: Scatterplot comparing DIA protein abundances to foldchanges for all 12 drugs combined. Lower left: Same as above only showing proteins identified as significant in DIA analysis (blue), or by both DIA and DDA (purple). Upper right: Scatterplot comparing DDA protein abundances to foldchanges for all 12 drugs combined. Lower right: Scatterplot comparing foldchanges between DIA and DDA differential analysis for all 12 drugs combined.
C) Venn diagram of proteins showing significant changes in RNA interaction under risdiplam treatment (adj. p<0.01) with an annotated involvement in RNA splicing.
D) Barplots comparing changes in RNA interaction for protein groups from C.
E) Scatterplot comparing foldchanges in protein-RNA interactions between risdiplam and ataluren treatment. Only proteins with an annotated involvement in RNA splicing are displayed, proteins with significant change under either treatment highlighted in yellow (adj. p<0.01, NBM testing).
F) Venn diagrams showing overlap of C2H2-type zinc-finger proteins between DNA and RNA-interacting proteomes. Left: All C2H2-type zinc-finger proteins derived under optimal irradiation conditions (RNA: 5 second UVEN 37 °C, DNA: 30 second UVEN 4 °C). Right: All C2H2-type zinc-finger proteins exclusively derived under optimal irradiation conditions (not present upon RNA: 5 second UV-bulb 37 °C, DNA: 30 second UVEN 37 °C).


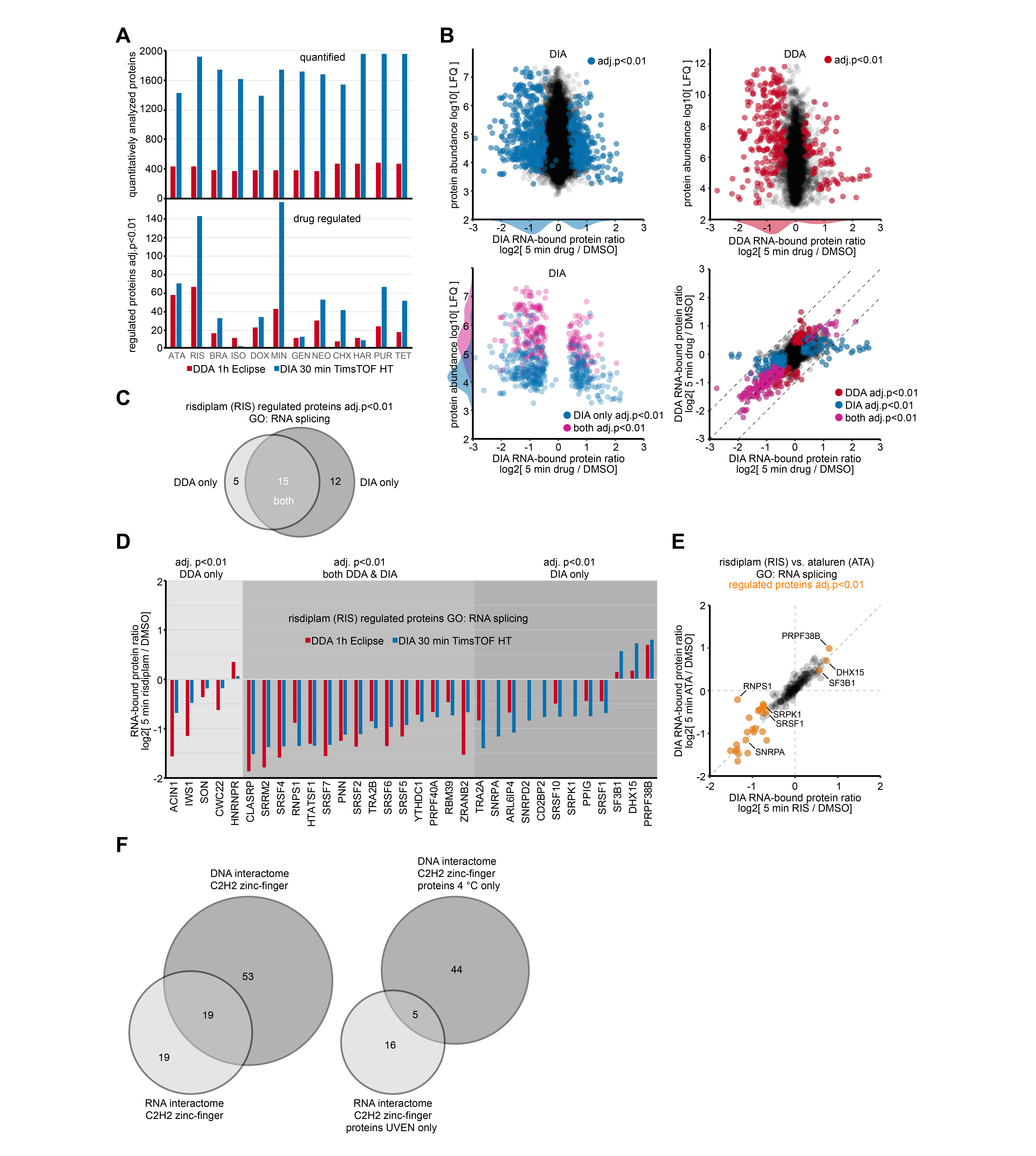


**
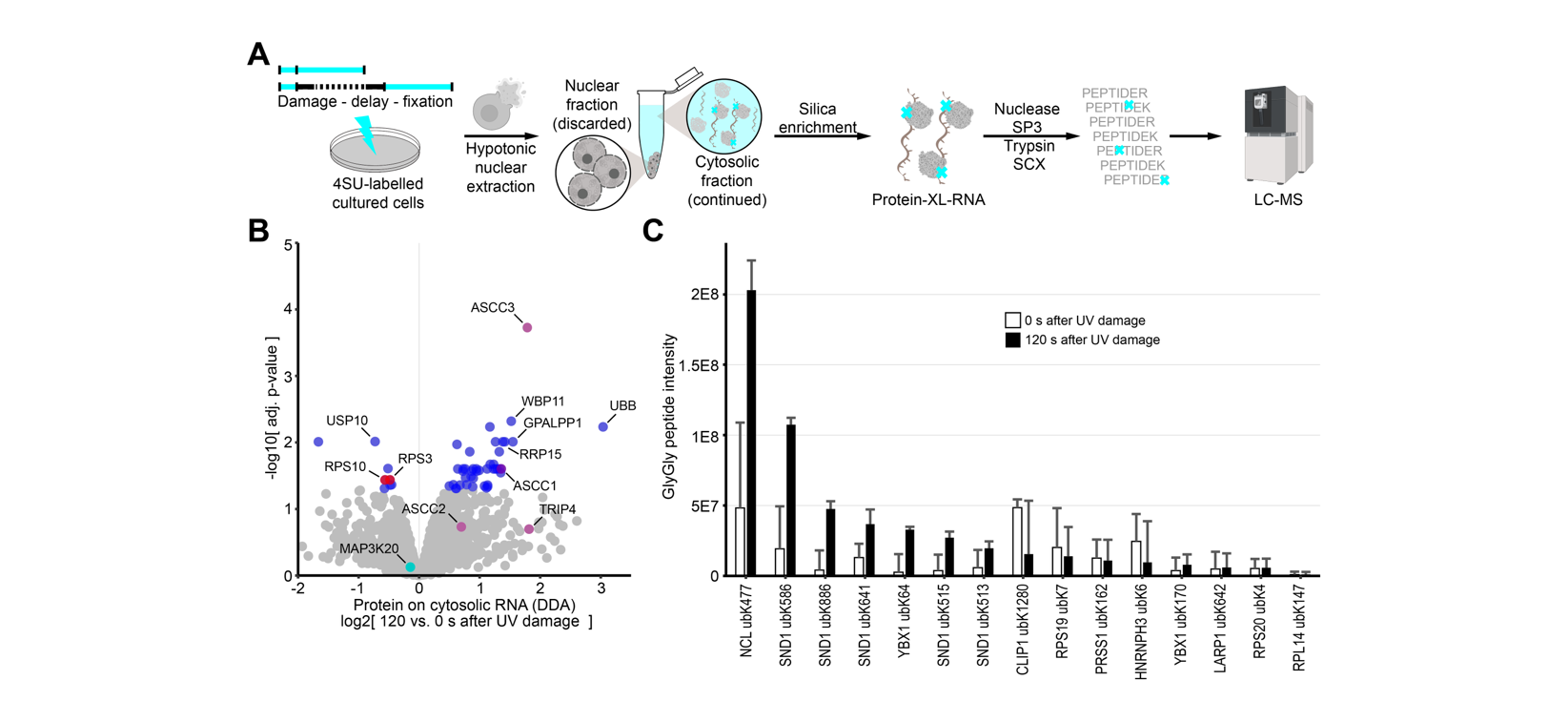
Figure S5: Extraction of protein-crosslinked cytosolic RNA and DDA analysis.**A) Workflow for the proteomic analysis of proteins photocrosslinked to cytosolic RNA (cytosolic XRNAX). Cells were irradiated as described in the text, and cytosolic fractions were separated from nuclei by hypotonic lysis. Cytosolic RNA was concentrated by isopropanol precipitation and purified using conventional silica spin columns. Co-isolated, photocrosslinked protein interactors were released by nuclease and trypsin digestion and subsequently quantified by LC-MS using either DDA (this figure) or DIA (see Figure 5). B) Volcano plot of the same samples as in Figure 5B, acquired by DDA rather than DIA. C) Bar plots comparing MS1 intensities of GlyGly-modified peptides (ubiquitination) detected in cytosolic RNA interactomes immediately after UVEN-induced damage (0 s after damage) or 120 s later. Error bars indicate one standard deviation of three biological replicates.

Figure S5 was partly created in BioRender. Trendel, J. (2026) https://BioRender.com/6mlk4ok
